# The chromatin landscape at the HIV-1 provirus integration site determines viral expression

**DOI:** 10.1093/nar/gkaa536

**Published:** 2020-06-29

**Authors:** Gerlinde Vansant, Heng-Chang Chen, Eduard Zorita, Katerina Trejbalová, Dalibor Miklík, Guillaume Filion, Zeger Debyser

**Affiliations:** Laboratory for Molecular Virology and Gene Therapy, Department of Pharmaceutical and Pharmacological Sciences, KU Leuven, Leuven, Flanders, Belgium; Center for Genomic Regulation (CRG), The Barcelona Institute of Science and Technology, Barcelona, Catalunya, Spain; Center for Genomic Regulation (CRG), The Barcelona Institute of Science and Technology, Barcelona, Catalunya, Spain; Institute of Molecular Genetics, Czech Academy of Sciences, Videnska, Prague, Czech Republic; Institute of Molecular Genetics, Czech Academy of Sciences, Videnska, Prague, Czech Republic; Center for Genomic Regulation (CRG), The Barcelona Institute of Science and Technology, Barcelona, Catalunya, Spain; University Pompeu Fabra, Barcelona, Catalunya, Spain; Laboratory for Molecular Virology and Gene Therapy, Department of Pharmaceutical and Pharmacological Sciences, KU Leuven, Leuven, Flanders, Belgium

## Abstract

HIV-1 persists lifelong in memory cells of the immune system as latent provirus that rebounds upon treatment interruption. Therefore, the latent reservoir is the main target for an HIV cure. Here, we studied the direct link between integration site and transcription using LEDGINs and Barcoded HIV-ensembles (B-HIVE). LEDGINs are antivirals that inhibit the interaction between HIV-1 integrase and the chromatin-tethering factor LEDGF/p75. They were used as a tool to retarget integration, while the effect on HIV expression was measured with B-HIVE. B-HIVE tracks insert-specific HIV expression by tagging a unique barcode in the HIV genome. We confirmed that LEDGINs retarget integration out of gene-dense and actively transcribed regions. The distance to H3K36me3, the marker recognized by LEDGF/p75, clearly increased. LEDGIN treatment reduced viral RNA expression and increased the proportion of silent provirus. Finally, silent proviruses obtained after LEDGIN treatment were located further away from epigenetic marks associated with active transcription. Interestingly, proximity to enhancers stimulated transcription irrespective of LEDGIN treatment, while the distance to H3K36me3 only changed after treatment with LEDGINs. The fact that proximity to these markers are associated with RNA expression support the direct link between provirus integration site and viral expression.

## INTRODUCTION

During infection with the human immunodeficiency virus type 1 (HIV-1), a DNA copy of the viral genome is inserted as provirus in a host chromosome by the viral integrase. Part of the proviruses enter a silent mode without gene expression rendering the virus invisible for the host immune system. These latent HIV proviruses mainly reside in long lived memory CD4^+^ T cells, that form a latent reservoir with an estimated half-life of 44 months (1–3). Although combination antiretroviral therapy (cART) suppresses plasma viral loads to undetectable levels, interruption of treatment allows latent proviruses to rebound (4,5). Therefore, cART is required lifelong and the latent reservoir is considered as the major barrier to cure HIV infection (6).

The predominant HIV-1 cure strategies aim to eliminate the latent reservoir (6,7). In order to achieve such cure, it is essential to understand the molecular determinants of HIV-1 latency. The impact of the chromatin environment on gene expression is only partially understood. Studies have shown that epigenetic silencing can contribute to HIV-1 latency; acetylated histones are generally associated with active provirus, while tri-methylation of H3K27 and H3K9 is associated with HIV silencing (8–13). Moreover, CpG methylation is known to maintain latency and resistance to reactivation (14,15). Less studied is the role of HIV-1 integration in the host genome and more specifically the impact of integration site selection on proviral gene expression. In 2001, the Verdin lab first postulated that the integration site affects transcription after observing highly variable expression levels between different transduced Jurkat clones (16). The Bushman lab reported that low-expressing proviruses integrate more often in genomic regions devoid of protein-coding genes and centromeric heterochromatin (17) and are less associated with DNase I sensitive sites, CpG islands and GC rich regions (18). Orientation of the provirus in the host gene was also suggested to play a role in HIV-1 expression (19–21).

The discovery of LEDGF/p75 as host factor of HIV integrase (IN) (22) led to a better understanding of the role of integration site selection. The integration step is catalyzed by IN (for a review see (23)). Lens-epithelium derived growth factor (LEDGF/p75), a transcriptional co-activator, tethers the viral pre-integration complex to transcriptionally active regions (24,25). LEDGF/p75 binds IN via its C-terminal Integrase Binding Domain (IBD) (22,26) and interacts with chromatin via its PWWP domain (27–29). The protein is known to recognize the H3K36me3 mark on nucleosomes (27–29). Depletion of LEDGF/p75 indeed shifts integration out of transcription units (30,31). In 2010 small molecule inhibitors of the interaction between IN and LEDGF/p75 were developed (32–37). These compounds, referred to as LEDGINs, are inhibitors of the well-defined interface between the IBD of LEDGF/p75 and the HIV-1 IN catalytic core (32–34,38). Potent LEDGINs with nanomolar activity inhibit HIV-1 IN allosterically (33,36–37). Later, it was found that LEDGINs also affect late stage replication steps; they disturb particle maturation by stimulating IN oligomerization, resulting in defective progeny virus (39–42). Vranckx *et al.* demonstrated that residual integrants after LEDGIN treatment are retargeted out of active genes to sites that are less transcriptionally active (31). At the same time, Chen *et al.* claimed that proviruses that integrate in proximity to endogenous enhancers display higher expression compared to those which are far from enhancers based on studies with barcoded HIV (B-HIVE) (43). Moreover, latent HIV was inserted ∼2-fold further away from endogenous enhancers than non-latent HIV (43). A similar conclusion was made by Miklik *et al.* who found that the proximity to active regulatory elements, particularly enhancers, correlates with stable proviral expression (44).

In the present study, we combined LEDGINs and B-HIVE to investigate the effect of integration site selection on HIV-1 transcription. B-HIVE is a technique that tags individual lentiviral vectors with a unique barcode of 20 random nucleotides (43,45). By simultaneously tracking each barcode in DNA and RNA of the same infected cells, transcription of proviruses can be correlated to the corresponding integration site (43,45). We here investigated the impact of LEDGIN-mediated retargeting on the chromosomal preference, epigenetic signature and viral expression of the residual provirus.

After treatment with LEDGINs, the distribution of HIV-1 integrants was less selective for gene-dense chromosomes (e.g. chromosomes 16, 17 and 19). LEDGINs retargeted the provirus towards silent genes and intergenic regions. They reduced overall RNA expression and increased the proportion of barcoded proviruses without expression. Finally, ‘no-expression’ sites obtained after treatment with LEDGINs were located further away from several epigenetic features associated with active transcription (H3K36me3, H3K79me2/3, H3K27ac, H3K4me1, RNAPII, H3K4me3, Med1, CBP). Interestingly, the distance of silent provirus to H3K36me3, the mark recognized by LEDGF/p75 increased after treatment with LEDGINs. In contrast, H3K27ac, H3K4me1, Med1 and CBP representative for (super-) enhancers, stimulated transcription regardless of LEDGIN-mediated retargeting. In conclusion, we show that the site of HIV-1 integration affects transcription by a combination of both general and LEDGF/p75-specific effects.

## MATERIALS AND METHODS

### Cell culture

SupT1 (provided by the National Institutes of Health reagent program, NIH, Bethesda, MD, USA) (46) and Jurkat cells (obtained from the cell collection of the Center for Genomic Regulation, CRG, Barcelona, Spain) were cultured in RPMI medium (GIBCO BRL) with 10% (v/v) fetal bovine serum (FBS, GIBCO) and 0.01% (v/v) gentamicin (GIBCO). HEK293T cells (generous gift from O. Danos, Evry, France) (47) were cultured in Dulbecco modified Eagle's medium (DMEM, GIBCO, Dublin, Ireland) with 5% (v/v) fetal bovine serum (FBS, GIBCO) and 0.01% (v/v) gentamicin (GIBCO). Cells were cultured at 37°C in a humidified atmosphere containing 5% CO_2_. All cells were verified to be mycoplasma free.

### Vector production

Linear polyetylenimine (PEI, Polysciences) was used to co-transfect HEK293T cells with the barcoded transfer plasmid pHCC1, Δ8.91 packaging plasmid and a vesicular stomatitis virus G (VSV-G) protein encoding plasmid to produce VSV-G-pseudotyped vectors. The barcoded plasmid pHCC1 was produced as described before (43). Six hours post transfection, cells were washed twice with phosphate buffered saline (PBS) to remove the excess of plasmid. Supernatant was collected 72 h post-transfection and filtered through a 0.45 μm pore membrane (Merck, Overijse, Belgium). The vector was concentrated using a Vivaspin with a 15–50 kDa cut-off column (Merck) and washed three times with PBS. Next, the vector was treated with 100 U/ml DNase (Roche Diagnostics, Vilvoorde, Belgium) for 1 h at 37°C and stored at −80°C.

### Transduction, DNA and mRNA purification

40 000 SupT1 and Jurkat cells were transduced with barcoded vector in the presence of different concentrations of LEDGIN CX014442 (33) (6.25, 15.62 and 31.25 μM) in a 96-well plate. The plates were centrifuged for 90 min at 1000 G. Twenty four hours after transduction, cells were washed twice with PBS and resuspended in medium containing LEDGIN CX014442 at the same concentrations of 6.25, 15.62 or 31.25 μM in a 96-well plate. GFP positive and negative cells were either sorted at day 4 and cultivated for 2 weeks, or directly cultivated for 2 weeks without sorting. While cultivating the cells, medium without LEDGINs was used. Sorting was performed on the S3e Cell Sorter (Bio Rad, Temse, Belgium). Genomic DNA and total RNA was isolated with an AllPrep DNA/RNA Mini Kit (Qiagen). The mRNA fraction was isolated from total RNA with an Oligotex mRNA Mini Kit (Qiagen).

### LEDGIN CX014442

LEDGIN CX014442 has been characterized previously using different techniques and viruses (33). Here we used a replication-deficient vector and calculated an IC_50_ of 6.5 μM based on inhibition of integrated copies in single round experiments. Thus the concentrations of 6.25, 15.62 and 31.25 μM correspond approximately to 1×, 2.5× and 5× IC_50_, respectively.

### Library preparation and sequencing

All DNA and RNA sequencing libraries were prepared as described before (43). DNA library preparation was performed via inverse PCR. mRNA was reverse transcribed to cDNA and amplified via barcode specific PCR. DNA integration site samples were sequenced with 76-bp paired-end Illumina next generation sequencing. RNA expression and DNA normalization samples were sequenced as 50-bp single reads on Illumina HiSeq.

### Flow cytometry and cell sorting

GFP expression was measured to determine transduction efficiency using a Guava^®^ Easycyte 5HT flow cytometer (Merck, Overijse, Belgium) with a 488 nm, 50 mW laser and 525/30 nm band pass filters. Before flow cytometry analysis, cells were fixed for 15 min at room temperature in 2% paraformaldehyde. GFP positive and negative cells were sorted 4 days after transduction by using the Bio-Rad S3™ cell sorter (Bio-Rad, Temse, Belgium) with a 488 nm, 100 mW laser. Prior to sorting, cells were washed and resuspended in PBS.

### qPCR

Total integrated HIV DNA was quantified using a nested real-time Alu-LTR qPCR (48). The first round PCR reaction mix consisted of 5 μl of genomic DNA, 10 μl of iQ supermix (Bio rad, Temse, Belgium), 0.5 μl of each primer (20 μM, Alu FW: TCCCAGCTACTGGGGAGGCTGAGG, Alu RV: TGCTGGGATTACAGGCGTGAG and HIV-1 LTR FW: GCTAACTAGGGAACCCACTGCTTA) and 3.5 μl of water. Cycling conditions for the first round PCR were 95°C for 10 min, followed by 15 cycles of 95°C for 30 s, 60°C for 40 s and 72°C for 3.5 min. 5 μl of the first-round product was added to a second round PCR mix containing 10 μl of iQ supermix, 0.5 μl of forward and reverse primer (20 μM, HIV-1 LTR FW: AGCTTGCCTTGAGTGCTTCAA, HIV-1 LTR RV: TGACTAAAAGGGTCTGAGGGATCT), 1 μl of probe (5 μM, 5′-FAM-TTACCAGAGTCACACAACAGACGGGCA-TAMRA-3′) and 3 μl of water. The second round PCR was performed in a LightCycler 480 (Roche Life Science, Vilvoorde, Belgium) for 5 min at 95°C, followed by 45 cycles of 95°C for 15 s, 60°C for 30 s and 72°C for 1 min. In parallel, a CCR5 qPCR was performed as previously described (49) to normalize for total input DNA. All samples were run at least in duplicate. Data were analyzed using the provided LightCycler 480 software.

### Bisulfite cytosine methylation analysis

SupT1 cells were transduced with OGH vector in the presence of varying concentrations of LEDGIN CX014442 (ranging between 3.12 and 25 μM). Two weeks post transduction, genomic DNA was extracted with the QIAamp DNA mini kit (Qiagen). 1 μg of gDNA was used for bisulfite conversion using the Epitect Plus DNA Bisulfite Kit (Qiagen) and eluted in a total of 20 μl of water. Bisulfite-treated DNA was amplified by PCR specific for the 5′ LTR in a 50 μl reaction mixture. PCR was performed with ∼75 ng of genomic DNA. Following primers were used: FW GGtAGAAtTAtAtAttAGGGttAGGGGTt, RV CACCCATCTCTCTCCTTCTAaCCTC. The sense primers contained T and the antisense primers A instead of C in positions complementary to non-methylable C (i.e. C in CpG dinucleotides). The reaction mixture contained 50 mM Tris–HCl (pH 9.2), 2.5 mM MgCl_2_, each dNTP at 200 μM, 320 nM each primer and 1 U of Platinum Taq Polymerase (Invitrogen), two mg of HotStart-IT Binding Protein (Affymetrix), and 1.5 μl of bisulfite-treated template DNA. Forty cycles were run under following conditions: 94°C for 20 s, 58°C for 50 s, and 72°C for 60 s. At least three primary PCRs were performed for each sample to exclude amplification of one template molecule. Non-converted DNA did not provide bands. Several non-template controls were included in each bisulfite PCR reaction. Amplification products were cloned in the pGEM-T-EasyVector System (Promega, Madison, WI, USA) and sequenced. Analysis was performed using the Quma (Quantification tool for Methylation Analysis) software (http://quma.cdb.riken.jp/). Only PCR clones with at least 95% conversion of cytosines outside CpGs were taken into account. When more converted molecules with identical sequences were obtained, only one was used for calculation of the methylated CpG percentage to minimize the bias originating from the preferential amplification of one molecule.

### RNA sequencing

We extracted total RNA from SupT1 and Jurkat cells, both untreated cells and cells cultured in the presence of 31.25 μM of LEDGIN CX014442, using the Aurum™ total RNA mini kit (Bio Rad). Sequencing libraries were prepared with the 3′ mRNA-seq library prep kit (Lexogen) and sequenced on Illumina HiSeq 4000.

### ChIP-sequencing

We generated ChIP-seq samples from untreated SupT1 and Jurkat cells, and from Jurkat cells cultured in the presence of 31.25 μM of LEDGIN CX014442 by using the Magna ChIP™ A/G Chromatin Immunoprecipitation Kit (Merck Millipore). Immunoprecipitations were performed with ChIP-grade antibodies against H3K36me3 (ab9050, Abcam) and H3K27ac (ab4729, Abcam). Samples were sequenced on Illumina HiSeq 4000.

### Gene enrichment analysis

We applied the function enrichKEGG involved in the R package clusterProfiler (50) to assay the enrichment Kyoto Encyclopedia of Genes and Genomes (KEGG) categories from less frequently HIV-targeted genes and genes harboring non-RNA expressing provirus with the false discovery rate (FDR) control represented by adjusted *P*-values. An adjusted *P*-value takes multiple statistic tests (individual P-values) into account in one entire dataset (51,52). We used the Benjamini–Hochberg procedure (53) to compute adjusted *P*-values for each enriched KEGG pathway in given gene sets. The Gene Ratio was calculated by taking the number of unique genes overlapping with those involved in a specific KEGG pathway (value k) divided by the number of unique genes overlapping with those in the collection of tested KEGG pathways (value *n*), based on the definition in the R package clusterProfiler. The equation for calculating Gene Ratio can thus be written as follows: GeneRatio = *k*/*n*. The output genes (value *k*) from the enriched pathways with significant adjusted *P*-values were annotated aside the node shown in the cnetplots.

### Quantification and statistical analysis

GraphPad Prism version 7.00 was used for statistical analysis (GraphPad Software, La Jolla, CA, USA, www.graphpad.com). Differences in distribution of integration sites across chromosomes and different genome categories were assessed by the Chi square test. The non-parametric Kruskal–Wallis with Dunn's multiple comparison test was used to compare distances of integration sites to certain features. In case other tests were used, the information is specified in the main text and figure legends.

### Bioinformatic analyses

ChIP-seq reads were mapped on GRCh37/hg19 with BWA-MEM with default parameters and a minimum mapping quality of 20. The targets were identified with Zerone v1.0 (54) with options ‘–list-output’ and ‘–confidence 0.99’.

Sequencing reads from Jurkat and SupT1 mRNA were mapped to Ensembl cDNA assembly GRCh37 (release 75) with kallisto (55) 46 with options ‘–single’ (single-end mode), ‘–bias’ (sequence bias correction, ‘–s300’ (fragment length 300 nucleotides) and ‘–l100’ (s.d. 100 nucleotides). The counts of the different isoforms were summed, thus generating a total count per gene copy in transcripts per million.

Identification of barcodes and HIV integration sites was performed as described before (43). The human genome was partitioned into six types: active genes, silent genes (genes refer to only protein-coding genes), active promoters, enhancers, intergenic regions and repeats. Active genes were defined as the 60% most expressed. Active promoters were defined as the regions spanning 5000 bp centered on the transcription start sites of active genes. Insertions were considered to be in the vicinity of an enhancer if their mapped location was within 2500 bp of a H3K27ac-enriched region. Insertions close to enhancers were in the enhancer category even if they were inserted inside a gene or a promoter. Genomic regions with bwa mappability score <20 were considered repeats and repeat classes were determined by classifying the raw FASTQ sequence with RepeatMasker. The rest of the genome was classified as intergenic.

### Data and code availability

All data-processing steps are documented in a Docker virtual machine available at https://github.com/ezorita/bhive. The datasets generated during this study are deposited at Gene Expression Omnibus (GEO), series GSE135295. Additional ChIP-Seq data for H3K36me3, H3K79me3, H3K27ac, H3K4me1, RNAPII, H3K4me3, H3K9me3 (GSE65687) (56), H3K79me2 (GSE60104) (57), Med1 (GSE59657) (58) and CBP (GSE17954) (59) were downloaded from GEO.

## RESULTS

### LEDGINs reduce the chromosomal bias of HIV integration

We have previously shown that LEDGIN treatment inhibits HIV-1 integration and that residual integrants are more often in a transcriptionally inactive state that is refractory to reactivation (31). Still, it is not clear whether the nature of the chromatin landscape surrounding the insertion site of those retargeted viruses can explain their latent state. We thus applied the B-HIVE technology (43,45) to track individual barcoded viruses retargeted by LEDGINs on a genome-wide scale.

**Figure 1.**
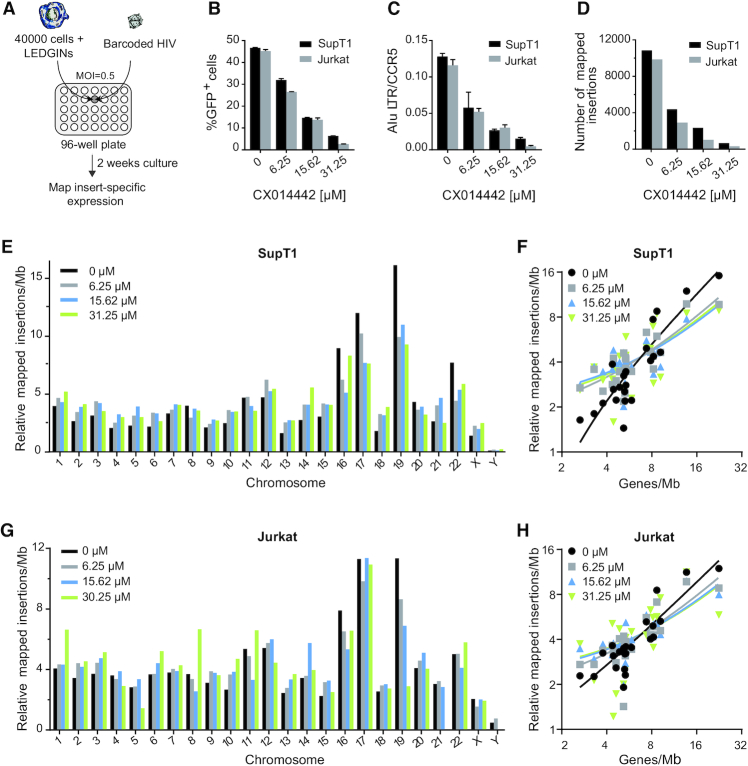
LEDGINs inhibit integration and reduce chromosomal preference. (**A**) 40 000 Jurkat or SupT1 cells were transduced in a 96-well plate with barcoded HIV in the presence of varying concentrations of LEDGIN CX014442. Cells were cultured for two weeks before DNA and RNA were extracted. (**B**) The percentage of GFP positive cells two weeks post transduction of SupT1 (black) and Jurkat (gray) cells in the presence of LEDGIN CX014442. Bars represent the average of flow cytometry measurement in duplicate with standard deviation. (**C**) The number of integrated copies two weeks post transduction, as determined by Alu-LTR nested qPCR on gDNA that was normalized to CCR5. Bars represent the average of qPCR in duplicate with standard deviation. (**D**) The number of retrieved integration sites in SupT1 and Jurkat cells. (E, G) Relative number of mapped insertions/Mb plotted for each chromosome in SupT1 (**E**) and Jukat (**G**) cells whereby the sum of all chromosomes per condition is 100. Different colors represent different concentrations of LEDGINs added during transduction. (F, H) XY-plot showing the relative number of mapped insertions/Mb (y-axis, log_2_ scale) over the gene density of each chromosome (x-axis, log_2_ scale) in SupT1 (**F**) and Jurkat (**H**) cells. Thus, each value on the x-axis corresponds to a certain chromosome (see also Supplementary Table S1 for gene densities) and to four y-values, one for each condition. The lines are the result of regression analysis (see also Supplementary Table S2). Two experiments were performed in Jurkat cells and two in SupT1 cells. Results are shown for one representative experiment, referred to as experiment A, in which SupT1 and Jurkat cells were transduced in parallel. GFP; Green Fluorescent Protein, Mb; megabase.

In this study, we adapted the transduction process used in the B-HIVE technology (Figure 1A). The improved method allows us to shorten the cultivation time needed to acquire sufficient genomic material and to retrieve at least four times more high-confidence insertion sites in the condition without LEDGIN compared to the previous method (43,45). Briefly, we transduced 40 000 SupT1 and Jurkat T cells with barcoded vector expressing GFP driven by the LTR promoter in the presence of 6.25, 15.62 or 31.25 μM of LEDGIN CX014442 in a 96-well plate (Figure 1A). Transduced cells were collected two weeks post transduction to map insert-specific expression. Inhibition by LEDGIN CX014442 was confirmed by flow cytometry (Figure 1B) and Alu-LTR qPCR (Figure 1C) 14 days post transduction in both SupT1 and Jurkat cells. Both the number of integrated copies and the percentage of GFP positive cells decreased with increasing concentration of LEDGIN CX014442. While on average 10 000 insertion sites were retrieved in the control samples, only 500 were obtained in cells treated with 31.25 μM of LEDGIN (Figure 1D).

HIV-1 prefers to integrate in chromosomes 16, 17 and 19 as these chromosomes have a high gene density (24). The obtained chromosomal distribution of HIV-1 integration was in line with previous studies (24,60) and reproducible in both SupT1 (Figure 1E) and Jurkat cells (Figure 1G). Treatment with LEDGIN CX014442 during infection significantly altered the chromosomal distribution as determined by the Chi-square test (*P*< 0.0001). The relative number of mapped insertions/megabase (Mb) decreased in chromosome 19 and to a lesser extent in chromosome 16 and 17 upon addition of LEDGIN CX014442 (Figure 1E and G, Supplementary Table S1). In other less gene-dense chromosomes more variability was observed (Supplementary Table S1). The chromosomal distribution of integration sites positively correlated with gene density of the chromosomes (Figure 1F and H). The average slope of the regression line dropped from 0.58 and 0.77 insertions per gene in Jurkat and SupT1 controls, respectively, to 0.31 and 0.36 in Jurkat and SupT1 cells treated with 31.25 μM of CX014442 (Jurkat *P* = 0.0172, SupT1 *P*< 0.0001, ANCOVA; Supplementary Table S2). Altogether, these results show that LEDGINs retarget integration away from gene dense regions.

### LEDGIN treatment retargets insertion sites towards silent genes and intergenic regions

**Figure 2.**
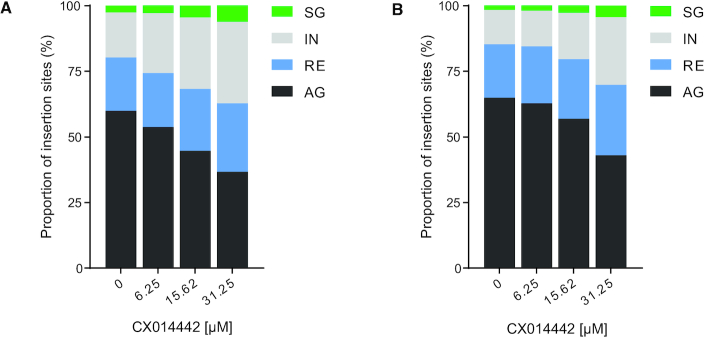
LEDGIN treatment retargets integration away from active genes. Integration sites retrieved in each condition in SupT1 (**A**) and Jurkat (**B**) cells were divided in four genomic categories and plotted as relative proportions: silent genes (SG), intergenic regions (IR), regulatory elements containing enhancers and promoters (RE) and active genes (AG).

We have previously shown that the genomic context and more specifically enhancer elements influence HIV-1 gene expression (43,44). In order to investigate whether LEDGINs can alter HIV-1 expression by retargeting insertion sites towards genomic regions that disfavor HIV expression, we first analyzed integration sites relative to some genomic features. We partitioned the genome into four types including active (protein-coding) genes (AG), silent (protein-coding) genes (SG), regulatory elements (RE) and intergenic regions (IR) based on SupT1 and Jurkat cell mRNA sequencing. Although the gene expression profile differed between SupT1 and Jurkat cells, treatment with 31.25 μM of CX014442 did not affect gene expression (Supplementary Figure S1). Since the proportion of promoter regions in the genome is limited, regions of active promoters and enhancers were combined as regulatory elements in this study. After calculating the proportion of integration sites in each category, we found that more proviruses were retargeted to SG and IR upon addition of increasing concentrations of LEDGIN CX014442 in SupT1 and Jurkat cells (*P*< 0.0001, Chi square test) (Figure 2). We further characterized the IR containing HIV integration sites and found that short and long interspersed nuclear elements (SINE and LINE), and retrotransposons were most frequently targeted. Addition of LEDGINs did not significantly alter the distribution of HIV within different types of IR (Supplementary Figure S2).

Next, we evaluated the effect of LEDGIN treatment on the type of genes that were targeted by applying the R package clusterProfiler on enriched pathways based on Kyoto Encyclopedia of Genes and Genomes (KEGG) (50,61–63). We retrieved the HIV-targeted genes lost after increasing the concentration of LEDGINs in SupT1 and Jurkat cells, referred to as ‘less frequently targeted genes’ (Supplemental results, Figures S3 and S4, Tables S3 and S4). The majority of these genes are highly transcribed genes (Supplementary Figures S3A and S4A). The endogenous gene expression of less frequently targeted genes after treatment with 31.25 μM of CX014442 was most affected. However, the change in endogenous gene expression was not consistent among genes involved in the same pathway (Supplementary Figures S3E, G and S4E, G).

### LEDGIN treatment increases the distance of integration sites to H3K36me3

**Figure 3.**
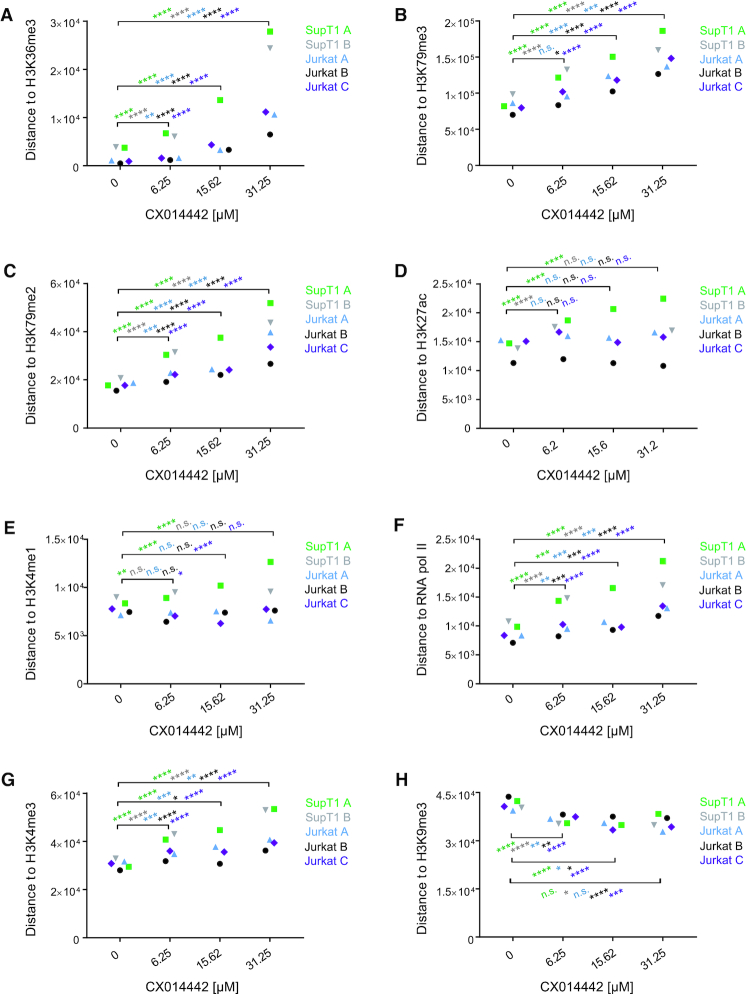
LEDGIN treatment retargets integration away from H3K36me3 in SupT1 and Jurkat cells. The median distance in base pairs (bp) between the integration site and certain features is plotted for two experiments in SupT1 cells and three in Jurkat cells. Panels A-H plot the distance to: (**A**) H3K36me3, (**B**) H3K79me3, (**C**) H3K79me2, (**D**) H3K27ac, (**E**) H3K4me1, (**F**) RNAPII, (**G**) H3K4me3 and (**H**) H3K9me3 (See also Supplementary Table S3 for explanation of different markers). Statistical significance was calculated by the Kruskal–Wallis test, * *P*< 0.05, ** *P*< 0.01, *** *P*< 0.001, **** *P*< 0.0001. RNAPII; RNA polymerase II.

Next, we plotted the distance of integration sites to certain epigenetic features (overview of features in Supplementary Table S5). Of all features analyzed, integration occurred closest to H3K36me3, the epigenetic histone modification recognized by LEDGF/p75 (27,28) and a marker for actively transcribed genes (64). Integration located on average at 1 kb distance from H3K36me3 in control conditions, indicating that this is an important target for HIV integration. LEDGIN treatment significantly increased the distance to H3K36me3 in both SupT1 and Jurkat cells (*P*< 0.0001, Kruskal–Wallis test) (Figure 3A). We also observed a LEDGIN-mediated increase in distance to H3K79me3 and me2 (ranging between 15 and 100 kb in control conditions) (Figure 3B and C) that are associated with gene bodies. H3K79me3 is bound by Hepatoma-derived growth factor-related protein 2 (HRP-2) (65), a paralogue of LEDGF/p75 that can take over its targeting function (66) and is inhibited by LEDGINs as well (67). The distance to H3K27ac and H3K4me1 (Figure 3D and E), both associated with enhancers (68), was not significantly altered. Finally, LEDGINs slightly increased the distance to RNAPII and H3K4me3 (Figure 3F and G), associated with transcription start sites (TSS) and promoters (69), while integration occurred somewhat closer to the silent chromatin marks H3K9me3 (Figure 3H) and to a lesser extent to H3K27me3 (data not shown). All analysis were performed using online available ChIP-seq data from untreated Jurkat cells. We obtained similar results when using ChIP-seq data that we generated in house in LEDGIN treated Jurkat cells and in SupT1 cells (Supplementary Figure S5).

We compared our results with sequencing results in LEDGF/p75 depleted SupT1 and Jurkat cells (Supplementary Figures S6 and S7). Although depletion of LEDGF/p75 in both cell lines shifted integration in a qualitatively similar manner to treatment with LEDGINs, 31.25 μM of CX014442 shifted integration more than depletion of LEDGF/p75. Overall, these data confirm that LEDGINs retarget integration out of active genes, towards silent genes and intergenic regions, implying a manifest role of LEDGF/p75 in selecting gene regions for transcription.

As methylation of the HIV 5′ LTR restricts HIV reactivation and contributes to latency as shown in cell lines and in patient samples (14,15), we next investigated the effect of LEDGINs on CpG methylation. We performed bisulfite cytosine methylation analysis on DNA sequences from cells treated with varying concentrations of LEDGIN during transduction (Supplementary Figure S8). 1% of sequenced CpG dinucleotides in the 5′ LTR of the control sample was methylated. Methylation significantly increased up to 3.3% when treated with 12.5 μM of CX014442 (*P* = 0.014, Chi-square test). However, there was no clear dose-response effect since at a concentration of 25 μM LEDGIN no further increase in methylation was detected.

### LEDGIN treatment reduces viral RNA expression

**Figure 4.**
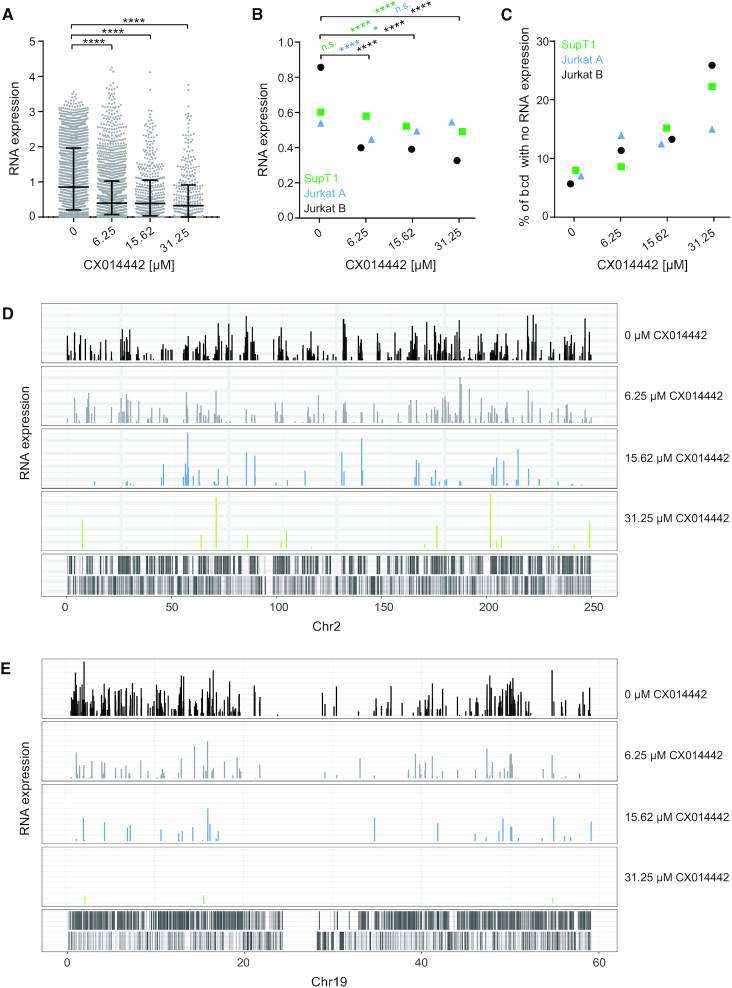
LEDGIN treatment reduces RNA expression. Two weeks post transduction in the presence of varying concentrations of LEDGIN CX014442, mRNA was extracted and reverse transcribed to cDNA to determine RNA expression. (**A**) Expression in Jurkat cells (experiment B) was determined using the B-HIVE method. The expression score is calculated for each unique barcode (dot) by normalizing the barcode counts in the RNA to the corresponding DNA counts. Error bars represent median and interquartile range. (**B**) Median RNA expression scores for two independent experiments in Jurkat cells (experiment A and B) and one in SupT cells (experiment A). (**C**) The percentage of DNA barcodes that did not show RNA expression was calculated relative to the total number of barcodes found in each condition in SupT1 and Jurkat cells. (**D**) Expression landscape of barcodes integrated in chromosome 2. Each bar represents an integration site and the height of the bar corresponds to the expression level. (**E**) Expression landscape of barcodes integrated in chromosome 19. Statistical significance was calculated by the Kruskal–Wallis test, **** *P*< 0.0001, *P< 0.05.

LEDGIN treatment was previously shown to increase HIV-1 latency using different reporter viruses in both cell lines and primary CD4^+^ T cells (31,48). In this study, we calculated RNA expression levels by using the B-HIVE method that is not limited to averaged readouts. mRNA was extracted from cells transduced in the presence of varying concentrations of LEDGIN CX014442 and reverse transcribed to cDNA. We were able to calculate expression levels of individual proviruses by normalizing cDNA counts of each barcode to the corresponding number of DNA counts. LEDGINs significantly reduced the median RNA expression per DNA copy in Jurkat cells (*P*< 0.0001, Kruskal–Wallis test) (Figure 4A). These results were confirmed in three independent experiments both in SupT1 and Jurkat cells (Figure 4B). We also retrieved many barcodes in the DNA without RNA expression. LEDGINs increased the percentage of these ‘no RNA’ barcodes up to 25% of total barcodes (*P*< 0.0001, Chi square test) (Figure 4C). We assume that these results indicate an overall reduction in HIV-1 RNA expression. Although we cannot entirely exclude the possibility that some of these non-expressing barcodes result from sequencing errors, we used these non-expressing barcodes to further characterize silent provirus. Interestingly, analysis of the integration landscape per chromosome revealed that some highly expressing barcodes persisted even after treatment with 31.25 μM of LEDGIN CX014442 (e.g. in chromosome 2, Figure 4D), while in some chromosomes LEDGINs reduced all expression (e.g. in chromosome 19, Figure 4E). Both high and low expressors were observed in several chromosomes and results varied between different experiments, indicating that this is not a chromosome specific effect.

Additionally, a B-HIVE experiment was performed in GFP-sorted SupT1 cells. Four days post transduction, GFP positive (+) and negative (–) cells were sorted and cultivated to perform DNA integration site sequencing. As expected LEDGINs reduced the total number of integration sites, although the relative proportion of integration sites retrieved in the GFP(–) population increased (Supplementary Figure S9A). Interestingly, the genomic distribution of integration sites from GFP(+), GFP(–) and unsorted SupT1 cells was comparable. Integration sites from all conditions, also from GFP(-) cells, were mostly found in active genes in the absence of LEDGINs (Supplementary Figure S9B). Yet, integrations obtained in GFP(–) cells seemed less associated with regulatory elements. LEDGINs had a similar retargeting effect in all conditions regardless of the sorting. Integration in active genes was reduced, while more provirus was found in intergenic regions and silent genes.

In conclusion, all methods show a reduction of RNA expression per provirus after LEDGIN treatment. Sorting of cells based on GFP expression provided no added value for further analysis of retargeting effects.

### HIV expression is influenced by both general and LEDGF/p75-specific epigenetic features

**Figure 5.**
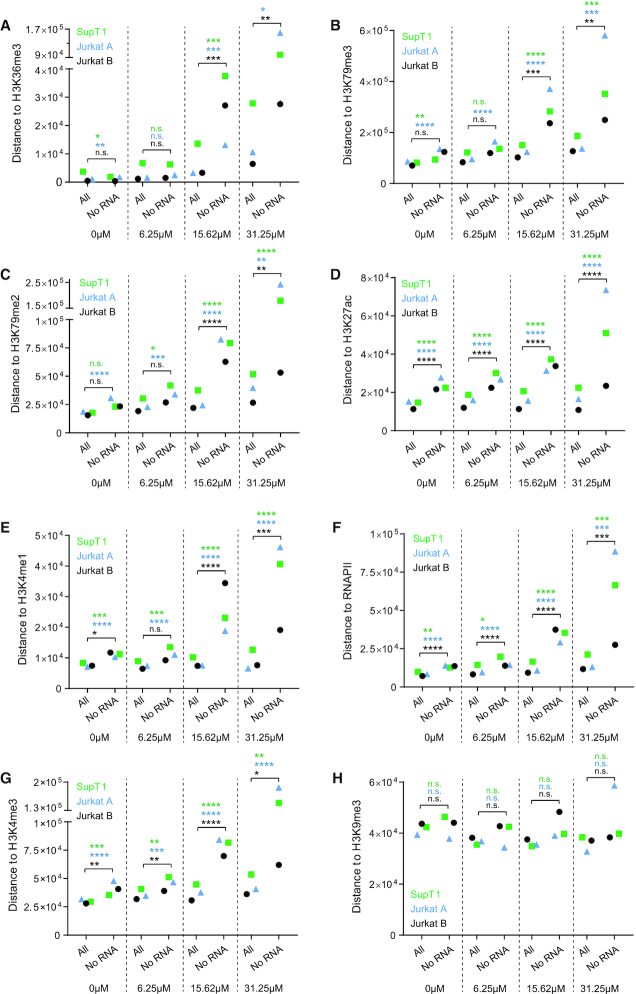
Silent barcoded provirus is targeted away from epigenetic features associated with active transcription. The distance in base pairs (bp) of integration sites to certain features was determined for either ‘all’ retrieved insertion sites or the ‘no RNA’ sites in each condition (0, 6.25, 15.62 or 31.25 μM of CX014442). The median distance (bp) is plotted on the y-axis for two independent experiments in Jurkat cells (experiment A blue, experiment B black) and one experiment in SupT1 cells (experiment A green). Panels A-H plot the distance to: (**A**) H3K36me3, (**B**) H3K79me3, (**C**) H3K79me2, (**D**) H3K27ac, (**E**) H3K4me1, (**F**) RNAPII, (**G**) H3K4me3 and (**H**) H3K9me3. Statistical significance was calculated by the Kruskal–Wallis test, * *P*< 0.05, ** *P*< 0.01, *** *P*< 0.001, **** *P*< 0.0001. bp; base pairs, RNAPII; RNA polymerase II.

**Figure 6.**
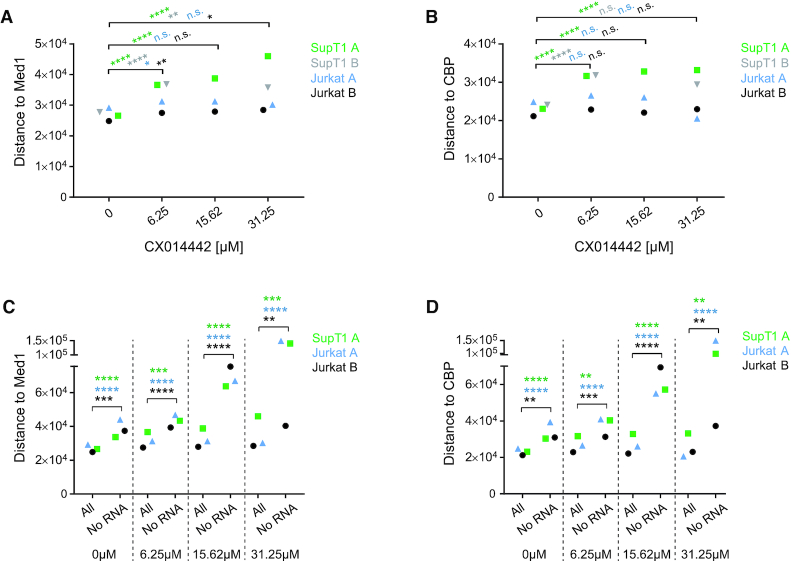
Effect of LEDGINs on super-enhancer markers Med1 and CBP. (A, B) Integration site analysis in SupT1 and Jurkat cells determined the distance in base pairs of HIV provirus to super-enhancer markers Med1 (**A**) and CBP (**B**) in each condition (0, 6.25, 15.62 or 31.25 μM of CX014442). The median distance (bp) is plotted on the y-axis for two independent experiments in Jurkat cells (experiment A blue, experiment B black) and two in SupT1 cells (experiment A green, experiment B gray). (C, D) The distance in base pairs (bp) of integration sites to Med1 (**C**) and CBP (**D**) was determined for ‘no RNA’ sites in each condition (0, 6.25, 15.62 and 31.25 μM of CX014442) and plotted next to ‘all sites’. The median distance (bp) is plotted on the y-axis for two independent experiments in Jurkat cells and one experiment in SupT1 cells. Statistical significance was calculated by the Kruskal–Wallis test, *P< 0.05, **P< 0.01, ***P< 0.001, ****P< 0.0001. bp; base pairs, Med1; Mediator 1, CBP; CREB-binding protein.

One major goal of this study was to investigate the link between integration sites and HIV-1 transcription. By using B-HIVE, we indeed show that LEDGINs retarget integration and reduce viral RNA expression. The following key question was to find out where these silent, non-RNA expressing proviruses are located. Therefore, we calculated the distances of these ‘no RNA’ barcoded proviruses (RNA expression = 0) to some epigenetic features and compared these barcodes with their transcriptionally active counterparts in each condition. The Kruskal-Wallis test was used to evaluate whether the distance of the ‘no RNA’ barcodes was altered compared to ‘all’ barcodes (Supplementary Figure S10). The median data for all three experiments in SupT1 and Jurkat cells revealed a good reproducibility of these distance plots (Figure 5). Interestingly, H3K36me3 affected barcode expression only after treatment with LEDGINs (Figure 5A). Although the distance to H3K36me3 did not change in cells without or with 6.25 μM of LEDGIN, the distance of DNA barcodes without RNA to all barcodes increased up to maximum eight and 15-fold when treating cells with 15.62 and 31.25 μM of CX014442 (Figure 5A, Supplementary Table S6). This result proves that LEDGF/p75 is a default determinant of integration site selection and that LEDGIN-mediated retargeting away from H3K36me3, as shown in Figure 3, negatively affects HIV-1 RNA expression. Non expressing proviruses were also 2- to 6-fold more separated from H3K79me3 and me2, associated with gene bodies, in cells treated with 15.62 and 31.25 μM of LEDGIN, respectively (Figure 5B and C). On the other hand, the median distance of ‘no RNA’ sites to the enhancer marker H3K27ac was approximately two times larger compared to ‘all’ sites in each condition, with or without LEDGINs (Figure 5D, Supplementary Table S4). These results indicate that enhancers affect transcription as reported (43,44). A similar, although less pronounced, effect was observed for another enhancer marker H3K4me1 (Figure 5E, Supplementary Table S6). RNAPII and H3K4me3, enriched near active promoters (69), stimulated transcription also in the absence of LEDGINs (Figure 5F and G, Supplementary Table S6). Treatment with LEDGIN CX014442 increased the distances between ‘all’ sites and ‘no RNA’ sites even more for all of these features. RNA expression was not affected by the distance to the silent chromatin mark H3K9me3 (69) (Figure 5H, Supplementary Table S6). We also investigated two markers associated with super-enhancers: Mediator 1 (Med1) and CREB-binding protein (CBP) (Figure 6) (70). As shown in Figure 6A and B, LEDGINs did not significantly change the distance of integration to the super-enhancer markers. Still, ‘no RNA’ provirus was located at increased distance to super-enhancers (1.3- to 7-fold increase compared to ‘all’ sites) (Figure 6C and D).

Next, we further characterized the non-RNA expressing proviruses by looking at the distribution across genic/non-genic regions and targeted genes. LEDGIN treatment retargeted integration away from active genes towards intergenic regions and silent genes. Interestingly, the observed retargeting effect seen for silent provirus (Supplementary Figure S11) was more pronounced compared to expressing proviruses (Figure 2). Secondly, we evaluated genes harboring non-RNA expressing provirus via the enrichment analysis based on KEGG categories (Supplemental results, Figure S12 and S13, Tables S7 and S8). Both in SupT1 and in Jurkat cells, we did not observe a direct link between endogenous gene expression and proviruses that do not transcribe RNA (Supplementary Figures S12 and 13).

In conclusion, these results indicate that the chromatin landscape at the site of integration determines HIV-1 RNA expression, independently of endogenous gene expression. Transcription depends on multiple determinants, among which H3K36me3, the recognition marker for LEDGF/p75, and enhancers, characterized by H3K27ac and H3K4me1, stand out. The positive correlation with proximity to (super-) enhancers is independent of LEDGF/p75, possibly explaining the presence of few residual high expressors after LEDGIN treatment. Indeed, opposite to non-expressing barcodes, the 10% highest expressing barcodes were found closer to H3K27ac and H3K4me1 (Supplementary Figure S14D and E), and to Med1 and CBP (data not shown). In line with results from Figure 5, high expressing sites were also closer to RNAPII and H3K4me3 (Supplementary Figure S14F and G). Of note, results obtained with high expressors were more variable due to a lower number of barcodes, especially in LEDGIN treated cells.

## DISCUSSION

The latent reservoir is the major hurdle for curing HIV and hence the main target for cure strategies (7,71). The extensively studied ‘shock-and-kill’ strategy aims to reactivate latent provirus followed by killing of reactivated cells by viral cytopathic effects or immune clearance (72,73). As such, this strategy attempts to eradicate the entire reservoir. This method is confounded by the high complexity of the latent reservoir and insufficient potency of presently available latency reversing agents (LRA) (74–76). It was shown that <5% of the reservoir reactivates upon stimulation (13). Moreover, reactivation of latent provirus is influenced by the site of integration indicating that a combination of multiple LRAs would be required (43). More recently, a strategy called ‘block-and-lock’ was proposed, that aims to permanently lock HIV provirus in a silent state unable to resume viral replication upon cART interruption (7,71). This latent state may be maintained by blocking HIV transcription with an inhibitor of trans-activator of transcription (Tat) (77–79). Alternatively, in order to permanently silence HIV, it might be feasible to retarget provirus to sites that are less susceptible to reactivation as shown with LEDGINs (31,48,80). Whatever strategy used, a better understanding of the role of the integration site in HIV latency is required. Here we used LEDGINs as a tool to study the effect of the chromatin landscape on viral RNA expression by retargeting HIV-1 integration. We used barcoded HIV-1 vectors to link single proviruses with chromatin features and transcription.

First, we confirmed that LEDGIN treatment during infection inhibits HIV-1 integration and retargets residual integrants. In line with previous results (31) the LEDGIN CX014442 retargeted integration out of active genes towards silent genes and intergenic regions (Figures 2 and 3). Integration frequency correlated with the gene density of chromosomes in agreement with previous results, but the chromosomal distribution was strikingly less selective for gene-dense chromosomes upon addition of LEDGIN (Figure 1, Supplementary Tables S1 and S2). LEDGINs profoundly affected the distance of HIV-1 provirus to H3K36me3, the recognition mark of LEDGF/p75 (27–29) (Figure 3): the distance increased from 1 kb on average in control samples to >10 kb in cells treated with 31.25 μM of CX014442. These results indicate that LEDGF/p75 is the predominant determinant of integration site selection. Although depletion of LEDGF/p75 altered the integration pattern in an analogous manner, the retargeting effect of 31.25 μM of LEDGIN CX014442 was stronger (Supplementary Figures S6 and S7). The stronger potency of LEDGIN compared to LEDGF/p75 depletion was documented before (31). Either high concentrations of LEDGINs reduce IN binding to LEDGF/p75 more than RNAi-mediated depletion of LEDGF/p75, or inhibition of the interaction between IN and HRP-2 plays a role in the overall phenotype. Hepatoma-derived growth factor-related protein 2 (HRP-2) (65), a paralogue of LEDGF/p75 that can take over its targeting function (66), is inhibited by LEDGINs as well (67). Of note, HRP-2 binds to H3K79me3, a feature found in transcribed genes (81–83) that was less favored for integration upon addition of LEDGINs. The fact that the distance to H3K79me3 was >70 kb in control samples and that knockdown of LEDGF/p75 affected the distance to this marker as well (Supplementary Figures S6 and S7), suggests that the role of HRP-2 may be rather small. Intriguingly, LEDGINs did not significantly alter the distance of provirus to the main enhancer markers H3K27ac and H3K4me1. Additionally, LEDGINs had a minor effect on the distance of HIV-1 provirus to RNAPII, H3K4me3 and H3K9me3, found at transcription start sites, promoters and transcriptionally silent regions, respectively (69). Finally, we investigated whether LEDGINs affect CpG methylation in the viral LTR promoter, as methylation was described to contribute to viral latency (14,15). In these experiments, LEDGINs had no major effect on methylation (Supplementary Figure S8). However, since in this experiment cells were not sorted based on GFP expression and only 10 to 20 sequences per condition were analyzed, these results might not be entirely representative for the latent population obtained after treatment with LEDGINs.

Next, we investigated the effect of LEDGIN-mediated retargeting on RNA expression. In agreement with our previous work (31), we showed that LEDGIN treatment reduced RNA expression per provirus in SupT1 and Jurkat cells (Figure 4). Consistently, a higher proportion of barcodes without RNA expression was detected in cells treated with LEDGINs. We investigated whether these silent barcodes are linked to certain chromatin features and found epigenetic markers that influenced HIV-1 transcription either in a LEDGF/p75-dependent or independent manner. After treatment with LEDGINs, silent barcodes were located at increased distance from H3K36me3, H3K79me2 and H3K79me3 compared to their transcriptionally active counterparts (Figure 5). These results indicate that inhibition of the interaction between HIV-1 IN and LEDGF/p75 affects HIV-1 RNA expression. Whether the interaction of LEDGF/p75 with nucleosomes directly activates HIV transcription remains unknown. Alternatively, they may target the provirus to chromatin that supports active transcription. Interestingly, ‘no RNA’ barcodes were located further away from markers associated with (super-) enhancers (H3K27ac, H3K4me1, Med1 and CBP) in all conditions, regardless of the presence of LEDGINs (Figures 5 and 6). This result is in agreement with previous findings (43,44). The influence of H3K27ac on expression was more pronounced than that of H3K4me1, possibly because H3K27ac is specifically associated with active enhancers, while H3K4me1 is also associated with poised enhancers (68,84). Finally, some other markers associated with active transcription like RNAPII and H3K4me3 were disfavored by silent viruses, also in the absence of LEDGINs. Although the median RNA expression decreased, some highly expressing barcodes persisted even in the presence of LEDGIN CX014442. The 10% highest RNA expressing barcodes were found closer to enhancers and to a lesser extent closer to RNAPII and H3K4me3 (Supplementary Figure S14). Altogether, these results prove (i) that the chromatin environment at the site of integration affects HIV transcription, (ii) that LEDGINs reduce transcription by retargeting provirus and that (iii) high RNA expression from residual integrants is due to their proximity to (super-) enhancers.

**Figure 7.**
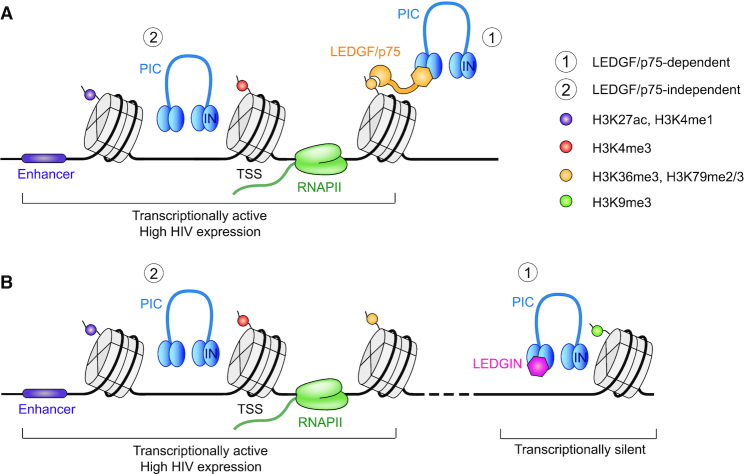
Model for HIV-1 integration and transcription. (**A**) HIV-1 integration is mainly determined by LEDGF/p75 binding to H3K36me3 in active genes. HIV-1 also integrates near enhancer regions characterized by H3K27ac and H3K4me1. Integration in these areas is associated with high RNA expression. (**B**) LEDGIN treatment retargets integration away from actively transcribed regions to silent genes and intergenic regions, resulting in provirus with lower RNA expression. However, some LEDGF/p75-independent integrations might still occur near regulatory elements, explaining few high residual expressors even in the presence of LEDGINs.

Based on these results we propose a model including LEDGF/p75-dependent and independent chromatin features determining HIV-1 RNA expression (Figure 7). LEDGF/p75 tethers HIV-1 to H3K36me3 in transcriptionally active regions. HIV-1 also integrates in proximity to enhancers in a LEDGF/p75 independent manner. Integration in these regions is associated with high HIV RNA expression. LEDGIN treatment retargets integration away from nucleosomes tagged with H3K36me3 by inhibiting the interaction between HIV-1 IN and LEDGF/p75. This LEDGIN-mediated retargeting negatively affects RNA expression. However, not all provirus is retargeted away from active regions by LEDGINs; some viruses still integrate in active regions or near enhancers, explaining the few high expressing barcodes persisting after treatment with LEDGINs. Those high expressors are associated with (super-) enhancers, RNAPII and promoters independently of LEDGF/p75. It is not clear whether integration near enhancers happens stochastically or is virus-induced. In fact, integration site selection has many determinants including nuclear import, host factors such as cleavage and polyadenylation specificity factor 6 (CPSF6) (85) and LEDGF/p75, and integrase recognition domains (86). After the pre-integration complex enters the nucleus through nuclear pore complexes (NPC), HIV integrates preferentially in the nuclear periphery in active chromatin close to the nuclear pore (87–93). Depletion of several NPC associated proteins (Nup98, Nup153, Transportin-3, RanBP2 and Tpr) hampers integration in gene dense regions (93–95). Interestingly, several studies reported that enhancers are frequently associated with nuclear pores (96–99). CPSF6 is an HIV cofactor that promotes nuclear entry via interaction with HIV capsid (85,100–102). Depletion and knockout of CPSF6 decreased integration in active genes (103–106). Additionally, HIV IN itself might affect integration site selection as it shows a weak preference for a conserved sequence logo at the site of integration (107–109). Moreover, HIV IN was shown to directly interact with chromatin via interaction with H4 amino-terminal tails (110).

In terms of HIV cure, this model suggests that the use of LEDGINs in a ‘block-and-lock’ cure strategy will affect residual HIV-1 expression but may not be sufficient to achieve complete repression of HIV-1 transcription. The present study hints at additionally blocking enhancer-stimulated transcription. Therefore, a combination of multiple latency promoting factors and transcription inhibitors might be required to silence all provirus. In addition, it might be interesting to target other integration site determinants like cofactors of nuclear import or chromatin remodelers to achieve a full remission. In future research, the role of HIV integration sites should also be investigated in clinically relevant models such as patient-derived cells. In addition to the B-HIVE method, it might be interesting to evaluate proviral sequences as data by Einkauf et al. suggest that intact proviral sequences in genomic regions associated with latency are selected over time (21).

## DATA AVAILABILITY

The datasets generated during this study are deposited at Gene Expression Omnibus (GEO), series GSE135295.

## Supplementary Material

gkaa536_Supplemental_Files

## Notes

Present address: Heng-Chang Chen, Institut de Génétique Humaine, Laboratoire de Virologie Moléculaire, UMR9002, CNRS, Université de Montpellier, Montpellier, France.

Present address: Guillaume Filion, Department of Biological Sciences, University of Toronto Scarborough, Canada.
