# Supplementary material for: The chromatin landscape at the HIV-1 provirus integration site determines viral expression
Source: Nucleic Acids Res. 2020 Jun 29;48(14):7801–17. doi: 10.1093/nar/gkaa536 (PMC7641320; doi:10.1093/nar/gkaa536)
Supplement: gkaa536_Supplemental_Files [file gkaa536_supplemental_files.zip › 20200419 all supplemental tables.pdf]

Change in relative number of insertions/Mb compared to 0 μM control

		SupT1 exp A				SupT1 exp B			Jurkat exp A				Jurkat exp B			
chromosome	genes/Mb	6.25 μM	15.62 μM	31.25 μM	χ^2	6.25 μM	31.25 μM	χ^2	6.25 μM	15.62 μM	31.25 μM	χ^2	6.25 μM	15.62 μM	31.25 μM	χ^2
13	2.65	0.93	1.12	1.10	***	0.65	0.79	*	0.34	0.89	1.25	*	-0.16	0.15	0.93	ns
18	3.29	1.47	1.37	2.07	****	0.94	1.17	ns	0.39	0.48	0.19	ns	0.05	-0.19	0.09	ns
4	3.77	0.45	1.18	0.93	***	0.63	0.26	ns	-0.24	0.28	-0.70	ns	0.53	-0.04	0.54	ns
8	4.36	-1.04	-0.26	-0.42	****	0.61	1.43	ns	-0.32	-1.13	2.96	ns	-0.55	-0.23	1.03	ns
21	4.43	1.37	2.03	-0.15	**	0.17	0.47	**	0.19	-0.20	-2.99	ns	0.04	-1.28	1.03	ns
5	4.62	0.87	1.66	0.74	****	2.09	-1.19	ns	0.05	0.54	-1.38	*	0.55	-0.04	0.03	ns
2	4.87	0.79	1.24	1.47	****	0.44	0.73	ns	0.97	0.74	1.11	*	0.45	0.42	-0.08	ns
X	5.19	0.85	0.58	1.09	***	0.39	0.69	ns	-0.50	-0.04	-0.11	ns	0.11	0.80	0.46	ns
3	5.2	1.24	1.08	0.39	***	0.57	0.66	***	0.72	1.02	1.42	ns	0.27	-0.19	0.60	ns
10	5.27	1.12	0.96	1.00	**	1.46	0.56	ns	0.99	1.17	2.03	***	0.42	0.91	0.08	ns
9	5.3	0.30	0.68	0.61	ns	0.85	1.29	ns	0.76	0.65	0.50	ns	-0.17	0.25	0.21	ns
15	5.33	1.13	1.05	1.02	*	0.66	1.19	*	0.90	1.01	0.25	ns	1.08	1.39	0.21	ns
7	5.37	0.31	0.78	0.74	ns	1.50	1.22	ns	0.24	0.09	0.48	ns	0.43	0.06	-0.87	ns
14	5.37	1.33	1.32	2.81	****	0.98	0.25	*	0.14	2.31	0.52	*	1.24	2.17	1.25	*
6	5.86	1.20	1.14	0.47	****	1.00	2.02	*	0.02	0.72	1.52	ns	-0.17	0.31	0.19	ns
12	7.37	1.51	0.53	0.73	*	0.93	0.61	ns	0.32	0.57	-0.98	ns	0.28	-0.67	-0.26	ns
1	7.86	0.68	0.32	1.23	ns	0.07	0.09	ns	0.27	0.25	2.55	ns	0.83	2.13	0.20	***
22	8.15	-3.29	-2.34	-1.84	****	-0.66	-0.40	ns	0.02	-0.91	0.77	ns	0.03	0.03	-0.86	ns
20	8.22	-0.67	-0.42	-1.10	*	-0.59	0.31	ns	0.49	1.00	-0.04	ns	1.58	0.65	-0.85	ns
16	8.67	-2.73	-3.84	-0.64	****	-2.61	-0.93	****	-1.38	-2.56	-1.33	****	-1.80	-2.45	-0.47	**
11	9.16	0.06	-0.74	-1.15	**	-0.38	0.64	ns	-0.48	-2.04	1.23	**	-0.38	-0.24	1.62	ns
17	13.68	-1.77	-4.30	-4.37	****	-3.11	-5.86	****	-1.47	0.07	-0.36	*	-1.89	0.19	-1.21	ns
19	22.53	-6.19	-5.14	-6.84	****	-6.90	-6.19	****	-2.70	-4.45	-8.43	****	-2.78	-3.95	-3.69	***

Table S1. Change in relative number of insertions/Mb compared to 0 μM control. The relative number of mapped insertions/Mb was calculated for each chromosome in all conditions (0, 6.25, 15.62 and 31.25 μM of CX014442). This table shows the difference in relative integration number between the 0 μM control and the different LEDGIN-treated conditions for each chromosome. Positive values indicate a relative increase in integration in a certain chromosome upon addition of LEDGINs, while negative values indicate less integration. Chromosomes are labeled in the first column and ranked based on their gene density (second column). Additionally, the results of Chi square tests per chromosome are shown for each experiment (* $p<0.05$, ** $p<0.01$, *** $p<0.001$, **** $p<0.0001$). Results are shown for two experiments in SupT1 cells and two in Jurkat cells. SupT1 experiment B was only done with 6.25 and 31.25 μM of LEDGIN CX014442.

Slope regression analysis

[CX014442]	SupT1 exp A	SupT1 exp B	Jurkat exp A	Jurkat exp B
0 µM	0.7663 ±0.07	0.8032 ±0.06	0.5814 ±0.06	0.5821 ±0.07
6.25 µM	0.4134 ±0.05	0.3793 ±0.05	0.3874 ±0.05	0.4145 ±0.06
15.62 µM	0.3365 ±0.04		0.3228 ±0.05	0.4043 ±0.08
31.25 µM	0.3632 ±0.07	0.388 ±0.05	0.3079 ±0.09	0.3752 ±0.08
P value	<0.0001	<0.0001	0.0172	0.1831

Table S2. Slopes obtained in regression analysis for relative number of insertions/Mb over gene density of chromosomes. Regression analysis was performed for XY graphs plotting the relative number of integrations/Mb over gene density of each chromosome (see Figure 1F and 1H for XY plots). This table lists the resulting slopes for two experiments in SupT1 and two in Jurkat cells. SupT1 experiment B was only done with 6.25 and 31.25 µM of LEDGIN CX014442. The P values in the bottom row, obtained with ANCOVA, indicate that the slopes are significantly different in three of four experiments.

Enriched Pathways	Enriched pathways after treatment with 6.25 µM CX014442		Pathway Identifier
Endocytosis	4. Cellular Processes	4.1 Transport and catabolism	04144
Salmonella infection	6. Human Diseases	6.8 Infectious disease: bacterial	05132
Autophagy - animal	4. Cellular Processes	4.1 Transport and catabolism	04140
Protein processing in endoplasmic reticulum	2. Genetic Information Processing	2.3 Folding, sorting and degradation	04141
Choline metabolism in cancer	6. Human Diseases	6.1 Cancer: overview	05231
Sphingolipid signaling pathway	3. Environmental Information Processing	3.2 Signal transduction	04071
T cell receptor signaling pathway	5. Organismal Systems	5.1 Immune system	04660
Mitophagy - animal	4. Cellular Processes	4.1 Transport and catabolism	04137
Pancreatic cancers	6. Human Diseases	6.2 Cancer: specific types	05212
Homologous recombinaton	2. Genetic Information Processing	2.4 Replication and repair	03440

Enriched Pathways	Enriched pathways after treatment with 15,62 µM CX014442		Pathway Identifier
Ubiquitin mediated proteolysis	2. Genetic Information Processing	2.3 Folding, sorting and degradation	04120
Cellular senescence	4. Cellular Processes	4.2 Cell growth and death	04218
Regulation of action cytoskeleton	4. Cellular Processes	4.5 Cell motility	04810
Cell cycle	4. Cellular Processes	4.2 Cell growth and death	04110
Growth hormone synthesis, secretion and action	5. Organismal Systems	5.2 Endocrine system	04935
Spinocerebellar ataxia	6. Human Diseases	6.4 Neurodegenerative disease	05017
Choline metabolism in cancer	6. Human Diseases	6.1 Cancer: overview	05231
Phosphatidylinositol singaling system	3. Environmental Information Processing	3.2 Signal transduction	04070
ErbB signaling pathway	3. Environmental Information Processing	3.2 Signal transduction	04012
T cell receptor signaling pathway	5. Organismal Systems	5.1 Immune system	04660

Enriched Pathways	Enriched pathways after treatment with 31.25 µM CX014442		Pathway Identifier
PI3K-Akt signaling pathway	3. Environmental Information Processing	3.2 Signal transduction	04151
MAPK signaling pathway	3. Environmental Information Processing	3.2 Signal transduction	04010
TNF signaling pathway	3. Environmental Information Processing	3.2 Signal transduction	04668
Neurotrophin signaling pathway	5. Organismal Systems	5.6 Nervous system	04722
HIF-1 signaling pathway	3. Environmental Information Processing	3.2 Signal transduction	04066
Glioma	6. Human Diseases	6.2 Cancer: specific types	05214
Longevity regulating pathway	5. Organismal Systems	5.9 Aging	04211
Prolactin regulating pathway	5. Organismal Systems	5.2 Endocrine system	04917
EGFR tyrosine kinase inhibitor resistance	6. Human Diseases	6.12 Drug resistance: antineoplastic	01521
PI3K-Akt signaling pathway	3. Environmental Information Processing	3.2 Signal transduction	04151

Table S3. Enriched KEGG pathways of genes less frequently targeted by HIV in SupT1 cells after treatment with LEDGIN CX014442. Category of the pathway map and the pathway identifier of enriched KEGG pathways from the genes that are less frequently targeted by HIV after treatment with 6.25 µM, 15.62 µM and 31.25 µM of LEDGIN CX014442 in SupT1 cells.

Enriched Pathways	Enriched pathways after treatment with 6.25 µM CX014442		Pathway Identifier
Endocytosis	4. Cellular Processes	4.1 Transport and catabolism	04144
Regulation of action cytoskeleton	4. Cellular Processes	4.5 Cell motility	04810
Ubiquitin mediated proteolysis	2. Genetic Information Processing	2.3 Folding, sorting and degradation	04120
Cell cycle	4. Cellular Processes	4.2 Cell growth and death	04110
Autophagy - animal	4. Cellular Processes	4.1 Transport and catabolism	04140
MAPK signaling pathway	3. Environmental Information Processing	3.2 Signal transduction	04010
Yersinia infection	6. Human Diseases	6.8 Infectious disease: bacterial	05135
ErbB signaling pathway	3. Environmental Information Processing	3.2 Signal transduction	04012
Adherens junction	4. Cellular Processes	4.3 Cellular community - eukaryotes	04520
N-Glycan biosynthesis	1. Metabolism	1.7 Glycan biosynthesis and metabolism	00510

Enriched Pathways	Enriched pathways after treatment with 15,62 µM CX014442		Pathway Identifier
Human immunodeficiency virus 1 infection	6. Human Diseases	6.9 Infectious disease: viral	05170
Human cytomegalovirus infection	6. Human Diseases	6.9 Infectious disease: viral	05163
Cellular senescence	4. Cellular Processes	4.2 Cell growth and death	04218
Ubiquitin mediated proteolysis	2. Genetic Information Processing	2.3 Folding, sorting and degradation	04120
Autophagy - animal	4. Cellular Processes	4.1 Transport and catabolism	04140
T cell receptor signaling pathway	5. Organismal Systems	5.1 Immune system	04660
mRNA surveillance pathway	2. Genetic Information Processing	2.2 Translation	03015
ErbB signaling pathway	3. Environmental Information Processing	3.2 Signal transduction	04012
EGFR tyrosine kinase inhibitor resistance	6. Human Diseases	6.12 Drug resistance: antineoplastic	01521
Non-small cell lung cancer	6. Human Diseases	6.2 Cancer: specific types	05223

Enriched Pathways	Enriched pathways after treatment with 31.25 µM CX014442		Pathway Identifier
Human cytomegalovirus infection	6. Human Diseases	6.9 Infectious disease: viral	05163
Thyroid hormone signaling pathway	5. Organismal Systems	5.2 Endocrine system	04919
Insulin signaling pathway	5. Organismal Systems	5.2 Endocrine system	04910
Protein processing in endoplasmic reticulum	2. Genetic Information Processing	2.3 Folding, sorting and degradation	04141
Choline metabolism in cancer	6. Human Diseases	6.1 Cancer: overview	05231
FoxO signaling pathway	3. Environmental Information Processing	3.2 Signal transduction	04068
Autophagy - animal	4. Cellular Processes	4.1 Transport and catabolism	04140
Longevity regulating pathway	5. Organismal Systems	5.9 Aging	04211
Endometrial cancer	6. Human Diseases	6.2 Cancer: specific types	05213
Homologous recombinaton	2. Genetic Information Processing	2.4 Replication and repair	03440

Table S4. Enriched KEGG pathways of genes less frequently targeted by HIV in Jurkat cells after treatment with LEDGIN CX014442. Category of the pathway map and the pathway identifier of enriched KEGG pathways from the genes that are less frequently targeted by HIV after treatment with 6.25 µM, 15.62 µM and 31.25 µM of LEDGIN CX014442 in Jurkat cells.

Marker	Meaning
H3k36me3	Gene bodies. LEDGF/p75 binding site
H3K79me2	Gene bodies
H3K79me3	Gene bodies. HRP-2 binding site
H3K27ac	Enhancer
H3K4me1	Enhancer
H3K4me3	Promoter
RNAPII	Transcription
H3K9me3	Transcriptionally silent
H3K27me3	Transcriptionally silent
Med1	Super-enhancer
CBP	Super-enhancer

Table S5. Overview and explanation of epigenetic markers. The epigenetic markers used in this study and their meaning are listed. RNAPII; RNA polymerase II, Med1; Mediator 1, CBP; CREB-binding protein,

Fold increase in distance between ‘all’ and ‘no RNA’ sites

	SupT1 exp A				Jurkat exp A				Jurkat exp B			
Feature	0 μM	6.25 μM	15.62 μM	31.25 μM	0 μM	6.25 μM	15.62 μM	31.25 μM	0 μM	6.25 μM	15.62 μM	31.25 μM
H3K36me3	0.50	0.93	2.75	3.51	1.63	1.56	4.01	15.10	0.82	1.27	8.18	4.25
H3K79me3	1.15	1.12	1.88	1.88	1.57	1.73	3.00	4.25	1.77	1.43	2.30	1.97
H3K79me2	1.30	1.38	2.11	3.46	1.64	1.49	3.39	6.10	1.50	1.40	2.84	1.99
H3K27ac	1.52	1.61	1.80	2.27	1.82	1.68	2.00	4.43	1.91	1.87	2.99	2.17
H3K4me1	1.34	1.52	2.26	3.21	1.45	1.49	2.50	7.05	1.57	1.43	4.65	2.51
RNA pol II	1.29	1.38	2.14	3.13	1.68	1.51	2.72	6.77	1.92	1.69	4.02	2.35
H3K4me3	1.20	1.26	1.83	2.77	1.51	1.34	2.24	4.58	1.45	1.23	2.27	1.71
H3K9me3	1.09	1.20	1.14	1.04	0.96	0.93	1.10	1.79	1.01	1.12	1.29	1.03

Table S6. Fold increase in distance between ‘all’ and ‘no RNA’ sites. The distance in base pairs (bp) of integration sites to certain features was determined for either ‘all’ retrieved insertion sites or the ‘no RNA’ sites in each condition (0, 6.25, 15.62 and 31.25 μM of CX014442). Next, the fold increase in median distance between ‘all’ and ‘no RNA’ sites was calculated and plotted. Values greater than ‘1’ indicate that ‘no RNA’ sites are located at increased distance to the feature compared to ‘all’ sites. Results are shown for one experiment in SupT1 cells and two in Jurkat cells.

Enriched Pathways	Enriched pathways in control condition (0 µM)		Pathway Identifier
AMPK signaling pathway	3. Environmental Information Processing	3.2 Signal transduction	04152
MAPK signaling pathway	3. Environmental Information Processing	3.2 Signal transduction	04010
Autophagy - animal	4. Cellular Processes	4.1 Transport and catabolism	04140
Sphingolipid signaling pathway	3. Environmental Information Processing	3.2 Signal transduction	04071
Neurotrophin signaling pathway	5. Organismal Systems	5.6 Nervous system	04722
Longevity regulating pathway	5. Organismal Systems	5.9 Aging	04211
Colorectal cancer	6. Human Diseases	6.2 Cancer: specific types	05210
Choline metabolism in cancer	6. Human Diseases	6.1 Cancer: overview	05231
Pancreatic cancer	6. Human Diseases	6.2 Cancer: specific types	05212
Endometrial cancer	6. Human Diseases	6.2 Cancer: specific types	05213

Enriched Pathways	Enriched pathways after treatment with 15,62 µM CX014442		Pathway Identifier
Axon guidance	5. Organismal Systems	5.8 Development and regeneration	04360
Chronic myeloid leukemia	6. Human Diseases	6.2 Cancer: specific types	05220
ErbB signaling pathway	3. Environmental Information Processing	3.2 Signal transduction	04012

Table S7. Enriched KEGG pathways of genes harboring non-expressing provirus in SupT1 cells. Category of the pathway map and the pathway identifier of enriched KEGG pathways from the genes targeted by HIV without RNA expression in SupT1 cells in control condition (0 µM) and treated with 15.62 µM of LEDGIN CX014442.

Enriched Pathways	Enriched pathways in control condition (0 µM)		Pathway Identifier
Viral carcinogenesis	6. Human Diseases	6.1 Cancer: overview	05203
mTOR signaling pathway	3. Environmental Information Processing	3.2 Signal transduction	04150
Thyroid hormone signaling pathway	5. Organismal Systems	5.2 Endocrine system	04919
Adherens junction	4. Cellular Processes	4.3 Cellular community - eukaryotes	04520
T cell receptor signaling pathway	5. Organismal Systems	5.1 Immune system	04660
Spinocerebellar ataxia	6. Human Diseases	6.4 Neurodegenerative disease	05017
Choline metabolism in cancer	6. Human Diseases	6.1 Cancer: overview	05231
EGFR tyrosine kinase inhibitor resistance	6. Human Diseases	6.12 Drug resistance: antineoplastic	01521
ErbB signaling pathway	3. Environmental Information Processing	3.2 Signal transduction	04012
Long-term potentiation	5. Organismal Systems	5.6 Nervous system	04720

Enriched Pathways	Enriched pathways after treatment with 6,25 µM CX014442		Pathway Identifier
Protein processing in endoplasmic reticulum	2. Genetic Information Processing	2.3 Folding, sorting and degradation	04141
Ubiquitin mediated proteolysis	2. Genetic Information Processing	2.3 Folding, sorting and degradation	04120
Autophagy - animal	4. Cellular Processes	4.1 Transport and catabolism	04140
RNA degradation	2. Genetic Information Processing	2.3 Folding, sorting and degradation	03018
Longevity regulating pathway	5. Organismal Systems	5.9 Aging	04211
Circadian rhythm	5. Organismal Systems	5.10 Environmental adaptation	04710
Lysine degradation	1. Metabolism	1.5 Amino acid metabolism	00310

Enriched Pathways	Enriched pathways after treatment with 31.25 µM CX014442		Pathway Identifier
Notch signaling pathway	3. Environmental Information Processing	3.2 Signal transduction	04330
Mitophagy - animal	4. Cellular Processes	4.1 Transport and catabolism	04137
Adherens junction	4. Cellular Processes	4.3 Cellular community - eukaryotes	04520
Thyroid hormone signaling pathway	5. Organismal Systems	5.2 Endocrine system	04919
Autophagy - animal	4. Cellular Processes	4.1 Transport and catabolism	04140
Phospholipase D signaling pathway	3. Environmental Information Processing	3.2 Signal transduction	04072
Wnt signaling pathway	3. Environmental Information Processing	3.2 Signal transduction	04310

Table S8. Enriched KEGG pathways of genes harboring non-expressing provirus in Jurkat cells. Category of the pathway map and the pathway identifier of enriched KEGG pathways from the genes targeted by HIV without RNA expression in Jurkat cells in control condition (0 µM), and treated with 6.25 µM and 31.25 µM of LEDGIN CX014442.
