# Supplementary material for: The chromatin landscape at the HIV-1 provirus integration site determines viral expression
Source: Nucleic Acids Res. 2020 Jun 29;48(14):7801–17. doi: 10.1093/nar/gkaa536 (PMC7641320; doi:10.1093/nar/gkaa536)
Supplement: gkaa536_Supplemental_Files [file gkaa536_supplemental_files.zip › 20200509 Supplementary results.docx]

**Supplemental results**

**Characterization of ‘less frequently targeted genes’**

We evaluated the effect of LEDGIN treatment on the type of genes that were targeted by applying the R package clusterProfiler on enriched pathways based on Kyoto Encyclopedia of Genes and Genomes (KEGG)^50,59–61^. We retrieved HIV-targeted genes lost after increasing the concentration of LEDGINs in SupT1 and Jurkat cells, referred to as ‘less frequently targeted genes’ (**Figure S3 and S4**). Those genes are mainly active genes, followed by regulatory elements and silent genes (**Figure S3A and S4A**). Thus, the majority of genes which are less frequently targeted by HIV in the presence of LEDGINS are highly transcribed genes. Even though many pathways were shared between SupT1 and Jurkat cells, they were not identical (**Figure S3A, B and S4A, B**). In SupT1 cells, the pathways involved in Genetic Information Processing, Environmental Information Processing, Cellular Processes, Organismal Systems and Human Diseases were unveiled (**Figure S3B**, **D and Table S3**). Pathways enriched in Human Diseases here include infectious diseases (*Salmonella* infection, 6.25 µM of CX014442), cancer (choline metabolism in cancer, 6.25 µM and 15.62 µM of CX014442; pancreatic cancers, 6.25 µM of CX014442; glioma, 31.25 µM of CX014442), neurodegenerative disease (spinocerebellar ataxia, 15.62 µM of CX014442) and drug resistance (EGFR tyrosine kinase inhibitor resistance, 31.25 µM of CX014442). It is worth to note that the genes that are no longer targeted by HIV at the highest dose of CX014442 (31.25 µM) are mainly involved in signal transduction, including the PI3K-Akt signaling pathway, the MAPK signaling pathway, the TNF signaling pathway and the HIF-1 signaling pathway (**Figure S**3**D and Table S3**). In Jurkat cells, we also detected gene sets enriched in the pathways involved in Metabolism, Human Diseases Genetic Information Processing, Environmental Information Processing, Cellular Processes, Organismal Systems and Human Diseases (**Figure S4B,** **D and Table S4)**. In comparison to the SupT1 cell line, two more gene sets involved in viral infectious disease, including HIV-1 infection (15.62 µM of CX014442), human cytomegalovirus infection (15.62 µM and 31.25 µM of CX014442) were detected in addition to the pathways involved in bacterial infectious disease, cancer and drug resistance (**Figure S4B, D and Table S4**). Few pathways involved in signal transduction were found when Jurkat cells were treated with the highest dose of CX014442 (31.25 µM) compared to SupT1 cells (**Figure S4D**).

Among returned KEGG pathways enriched mentioned above, we selected those with significant adjusted P-values (Wright, 1992) for each dose of CX014442 and illustrated the connection between the enriched pathways and our given gene sets (**Figure S3E, G and Figure S4E, G**). In every cnetplot, each yellow node which represents one enriched pathway is associated with the genes involved. The color code in each annotated gene corresponds to the fold change in logarithm of endogenous gene expression after cells treated by CX014442. We found that the fold change is higher when cells were treated with 31.25 µM of CX014442 (**Figure S3G and S4G**) compared to those treated with 6.25 µM (**Figure S3E and S4E**) and 15.62 µM (**Figure S3F and S4F**) of CX014442 in both cell lines. Fold change in endogenous gene expression is however not consistent among the genes involved in the same pathway (**Figure S3E, G and Figure S4E, G**).

**Characterization of genes harboring non-RNA expressing provirus**

We evaluated genes harboring non-RNA expressing provirus via the enrichment analysis based on KEGG categories (**Figure S12 and S13**). Enriched pathways were only retrieved when SupT1 cells were not treated and with 15.62 µM of CX014442 (**Figure S12B and C**). The majority of the pathways enriched in both experimental conditions are involved in signal transduction and cancer (**Figure S12B, C and Table S7**). Although the fold change of endogenous gene expression corresponding to each annotated gene involved in the pathways with significant adjusted P-values shown in the cnetplot displayed a minor magnitude in both scenarios (**Figure S12B and C**), no direct link of endogenous gene expression to proviruses with no RNA expression was observed here. Enriched KEGG pathways observed in Jurkat cells (**Figure S13B and C**) are relatively diverse compared to those found in SupT1 cells. We did not observe pathways when cells were treated with 15.62 µM of CX014442. In addition to the pathways involved in signal transduction and cancer, pathways involved in metabolism, genetic information processing, cellular processes, organismal systems and other types of human diseases were found as well (**Figure S13B, C and Table S8**). Due to low numbers of unique genes targeted by non-RNA expressing proviruses when treated with 31.25 µM of CX014442 (**Figure S13A**), there are few overlapping genes between the given gene set and the gene set involved in each retrieved pathway (two genes were found in each pathway) (**Figure S13D**). Similar to results obtained in SupT1 cells, we did not observe any direct link between the fold change of endogenous gene expression triggered by CX014442 and proviruses which do not transcribe RNA in Jurkat cells (**Figure S13E-G**). We observed independent KEGG pathways enriched by the genes targeted by non-RNA expressing proviruses in Jurkat cells treated with 6.25 µM (RNA degradation) and 31.25 µM (Autophagy - animal and Mitophagy - animal) of CX014442, respectively (**Figure S13F and G**).
