# Supplementary material for: The chromatin landscape at the HIV-1 provirus integration site determines viral expression
Source: Nucleic Acids Res. 2020 Jun 29;48(14):7801–17. doi: 10.1093/nar/gkaa536 (PMC7641320; doi:10.1093/nar/gkaa536)
Supplement: gkaa536_Supplemental_Files [file gkaa536_supplemental_files.zip › 20200510 All supplemental figures.pdf]

Figure S1. Comparative mRNA sequencing analysis in SupT1 and Jurkat cells.

A)

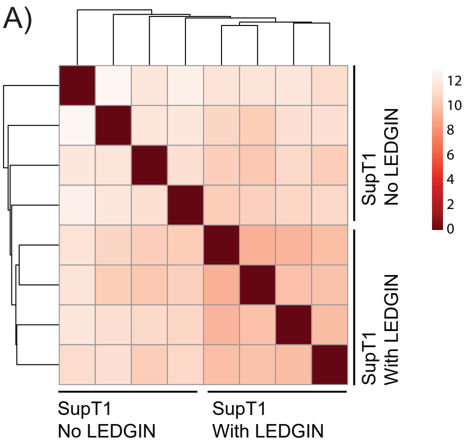

B)

id	baseMean	log2FoldChange	lfcSE	stat	padj
MT-RNR1	37242.50	-0.25	0.05	-4.8402	0.028349
NEAT1	1538.07	-0.33	0.07	-4.576	0.051783
RNU6-758P	56.84	-1.11	0.25	-4.4274	0.069471
KIF15	564.65	0.31	0.08	3.8817	0.52319
RPS16	4454.23	0.17	0.04	3.8467	0.52319
RPS17	2593.58	0.25	0.07	3.6647	0.90188
7SK	0.48	-0.74	2.35	-0.31488	0.99989
A1BG	1.44	-0.98	1.26	-0.77994	0.99989
A1BG-AS1	9.08	-0.27	0.53	-0.50578	0.99989
A2M-AS1	1.13	1.32	1.60	0.8248	0.99989
A2MP1	0.85	-1.91	2.13	-0.89582	0.99989
A4GALT	0.17	0.67	3.53	0.18913	0.99989
AAAS	39.47	0.27	0.25	1.0532	0.99989

C)

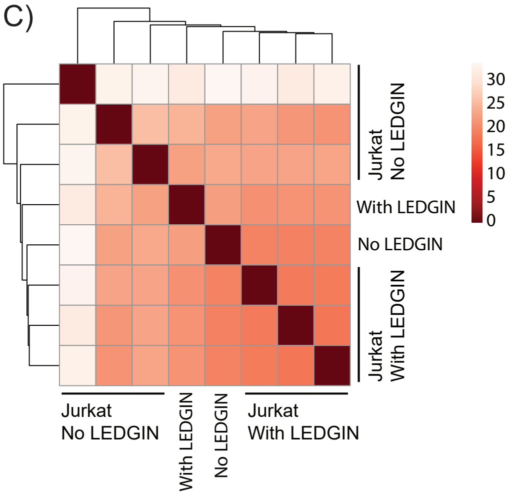

D)

id	baseMean	log2FoldChange	lfcSE	stat	padj
GCOM2	21.95	1.51	0.36	4.1479	0.76654
7SK	0.80	1.09	1.75	0.62153	0.99806
A1BG	2.58	0.14	1.04	0.13859	0.99806
A1BG-AS1	18.35	0.30	0.38	0.78417	0.99806
A2M-AS1	0.56	0.38	2.22	0.17121	0.99806
A2MP1	0.23	-0.51	3.29	-0.15582	0.99806
AAAS	97.44	-0.24	0.27	-0.87277	0.99806
AACS	126.86	0.14	0.19	0.72462	0.99806
AADAT	17.38	-0.25	0.39	-0.62955	0.99806
AAED1	30.21	0.02	0.31	0.069053	0.99806
AAGAB	212.29	0.08	0.14	0.5582	0.99806
AAK1	302.82	0.10	0.11	0.93322	0.99806
AAMDC	66.23	-0.07	0.25	-0.29582	0.99806

E)

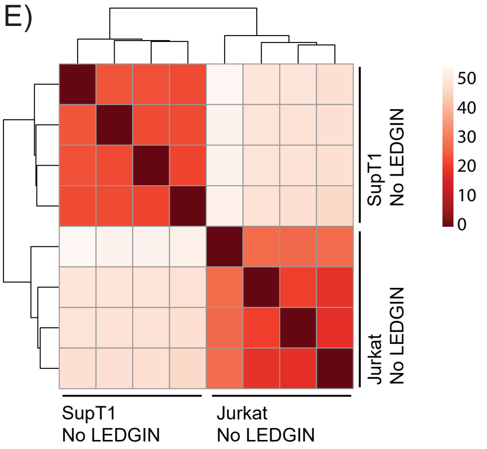

F)

id	baseMean	log2FoldChange	lfcSE	stat	padj
EPHB6	790.86	3.63	0.11	32.525	8.0046e-228
DNAJB1	1782.39	-5.06	0.17	-30.133	1.5561e-195
TRBC1	1752.28	-8.80	0.31	-28.199	3.4486e-171
RP11-144L1.4	290.71	4.69	0.17	27.789	2.5127e-166
NOTCH1	418.00	3.31	0.12	27.639	1.2807e-164
WNK2	293.73	5.11	0.19	26.64	6.7253e-153
ARL4C	1515.38	2.30	0.09	26.18	1.0854e-147
KIF3A	972.89	2.91	0.11	26.068	1.8128e-146
CYFIP1	315.39	4.00	0.16	24.804	1.5382e-132
TRBC2	1719.65	3.60	0.15	24.58	3.5684e-130
PDP1	320.45	3.32	0.14	24.549	6.9833e-130
GYPC	926.18	-8.00	0.33	-24.538	8.3566e-130
SMPD3	319.14	4.50	0.19	24.131	1.5523e-125

Figure S1. Comparative mRNA sequencing analysis in SupT1 and Jurkat cells. mRNA from SupT1 and Jurkat cells with or without addition of 31.25 μM of LEDGIN CX014442 was sequenced. For each condition four replicates were sequenced. The effect of LEDGINs was assessed in SupT1 cells (A,B) and Jurkat cells (C,D). Finally SupT1 and Jurkat cells without LEDGIN treatment were compared (E,F). Panels A,C and E: heatmap of the sample-to-sample distances. Panels B,D and F: top 13 of differential expressed genes. Padj is the p-value adjusted for multiple testing with the Benjamini-Hochberg, which controls false discovery rate.

Figure S2. Characterization of integration sites in intergenic regions.

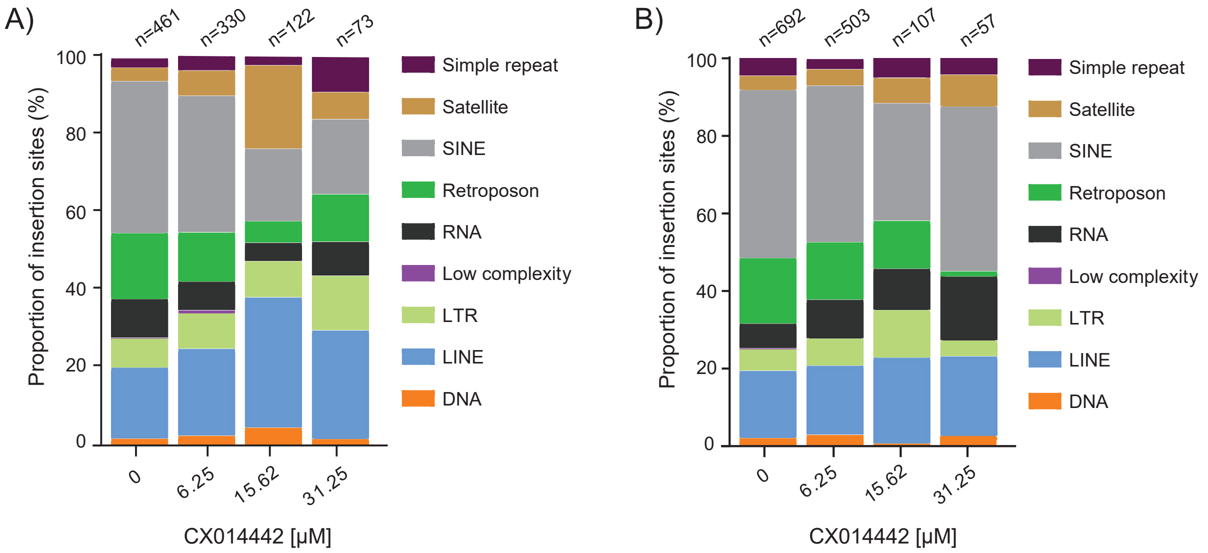

Figure S2. Characterization of integration sites in intergenic regions. The proportion of integration sites in different types of intergenic regions is calculated in SupT1 cells (A) and Jurkat cells (B) from experiment A.

Figure S3. Genes less frequently targeted by HIV-1 after treatment with LEDGINs in SupT1 cells.

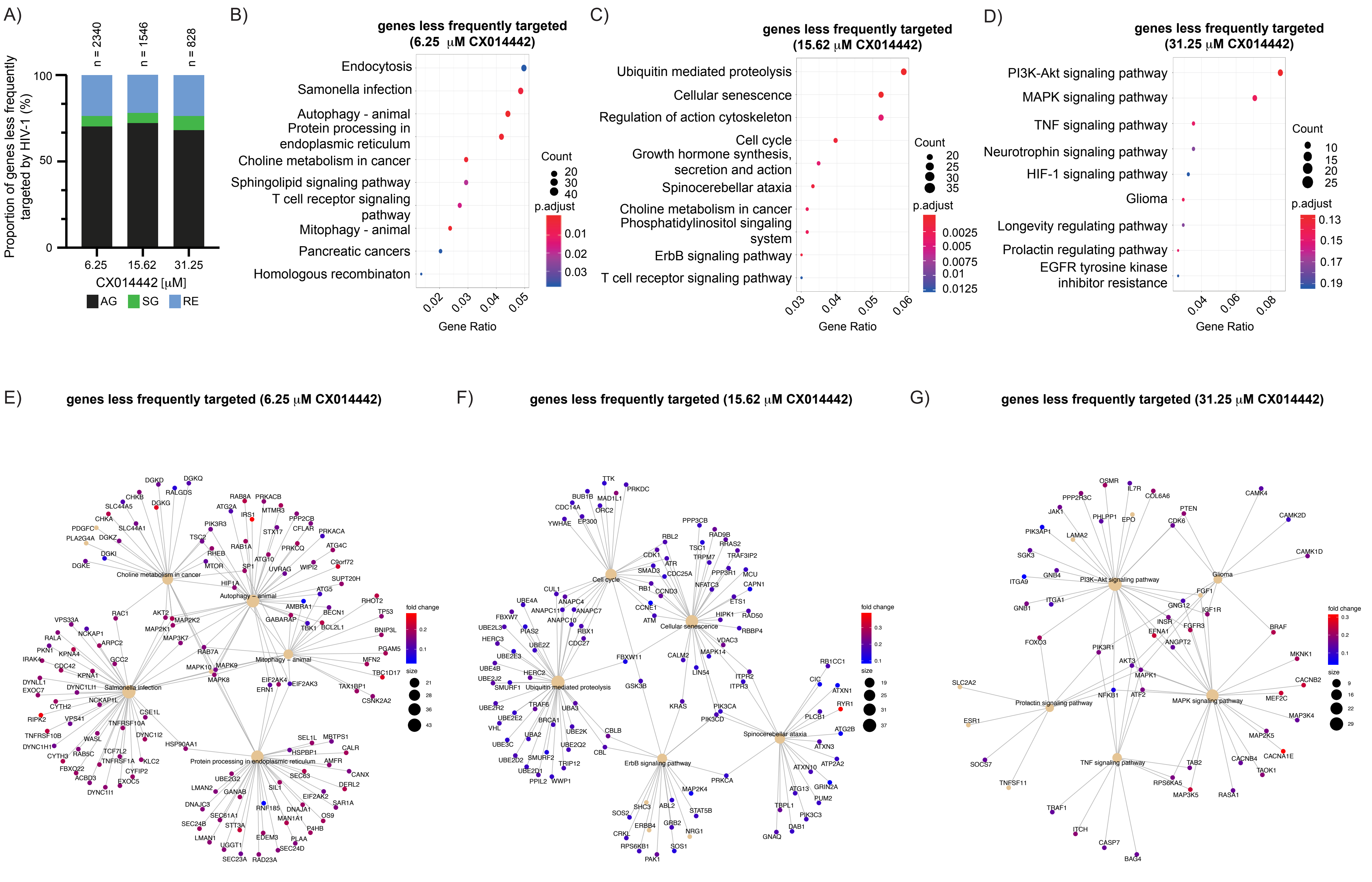

Figure S3. Genes less frequently targeted by HIV after treatment with LEDGINs in SupT1 cells. A) Proportion of genes that are less frequently targeted by HIV in different genome categories, after treatment with different concentrations of LEDGIN CX014442 (6.25, 15.62 or 31.25 μM) in SupT1 cells. AG: active genes; SG: silent genes; RE: regulatory elements. B-D) Enriched KEGG-defined pathways from the genes less frequently targeted by HIV after treatment with 6.25 μM (B), 15.62 μM (C) or 31.25 μM (D) of CX014442. The size of the circle indicates the number of less frequently targeted genes involved in each enriched pathway. The color of the circle indicates the adjusted P-value in each enriched pathway. E-G) The cnetplot illustrates the connection between the enriched pathways with significant adjusted P-values and the less frequently targeted genes after treatment with 6.25 μM (E), 15.62 μM (F) or 31.25 μM (G) of CX014442. The yellow node represents the selected enriched KEGG pathway. The size of the yellow node indicates the number of genes involved in each selected enriched pathway. The color code in each node aside the annotated gene corresponds to the fold change in logarithm of endogenous gene expression after treatment with LEDGIN CX014442.

Figure S4. Genes less frequently targeted by HIV-1 after treatment with LEDGINs in Jurkat cells.

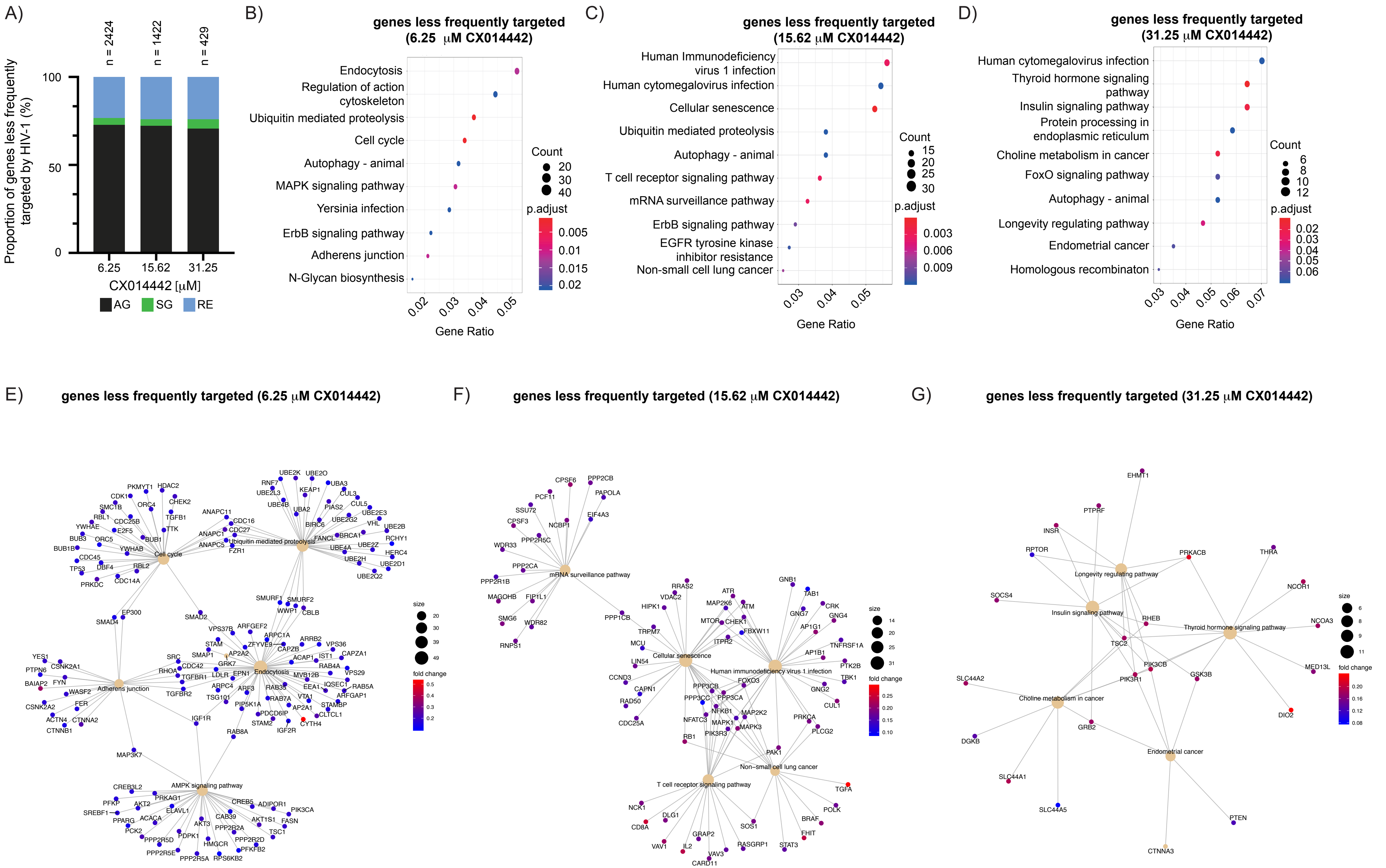

Figure S4. Genes less frequently targeted by HIV after treatment with LEDGINs in Jurkat cells. A) Proportion of genes that are less frequently targeted by HIV in different genome categories, after treatment with different concentrations of LEDGIN CX014442 (6.25, 15.62 or 31.25 µM) in Jurkat cells. AG: active genes; SG: silent genes; RE: regulatory elements. B-D) Enriched KEGG-defined pathways from the genes less frequently targeted by HIV after treatment with 6.25 µM (B), 15.62 µM (C) or 31.25 µM (D) of CX014442. The size of the circle indicates the number of less frequently targeted genes involved in each enriched pathway. The color of the circle indicates the adjusted P-value in each enriched pathway. E-G) The cnetplot illustrates the connection between the enriched pathways with significant adjusted P-values and the less frequently targeted genes after treatment with 6.25 µM (E), 15.62 µM (F) or 31.25 µM (G) of CX014442. The yellow node represents the selected enriched KEGG pathway. The size of the yellow node indicates the number of genes involved in each selected enriched pathway. The color code in each node aside the annotated gene corresponds to the fold change in logarithm of endogenous gene expression after treatment with LEDGIN CX014442.

Figure S5. Results using ChIP-seq from LEDGIN treated Jurkat and SupT1 cells.

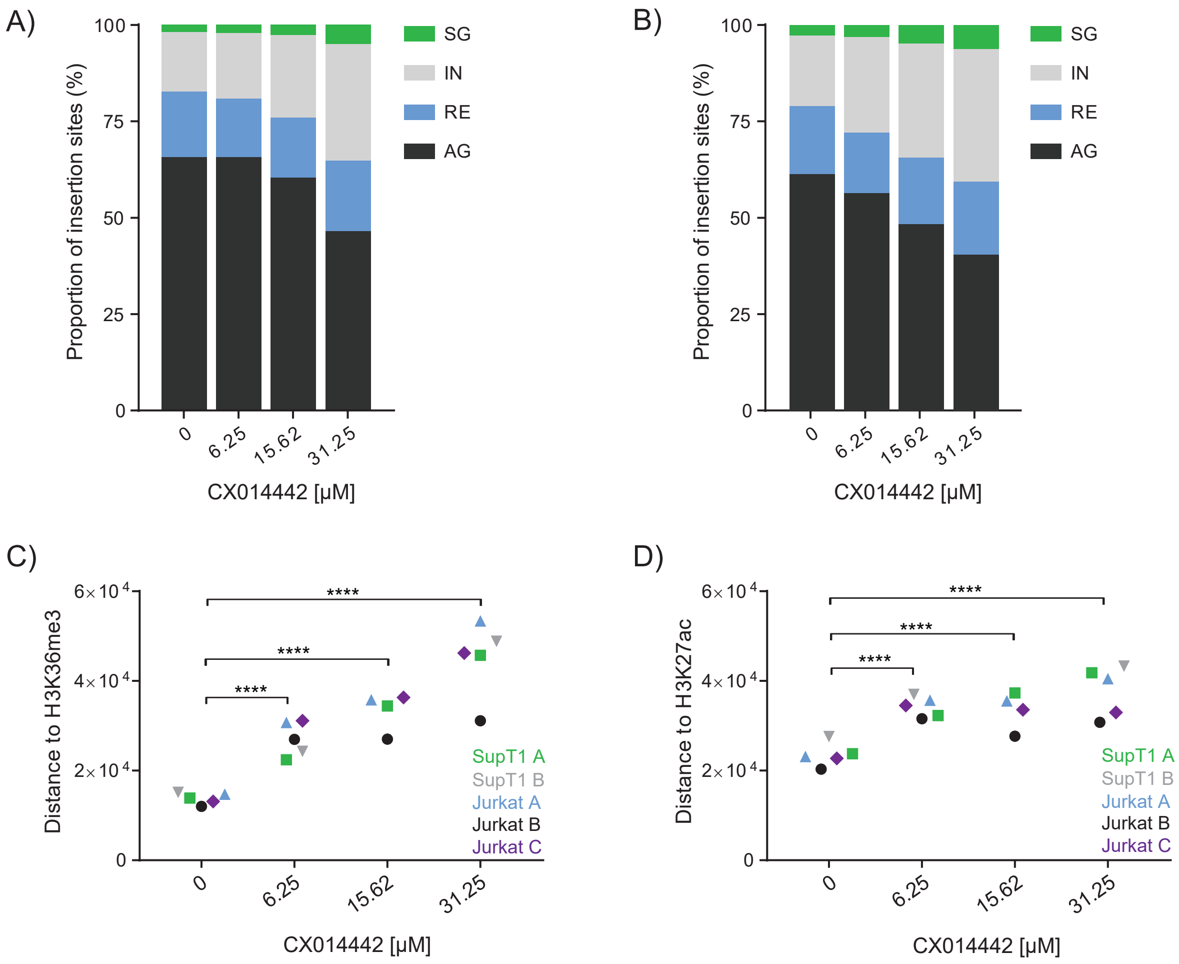

Figure S5. Results using ChIP-seq from LEDGIN treated Jurkat and SupT1 cells. ChIP-seq for H3K36me3 and H3K27ac was performed in Jurkat cells, untreated and treated with 31.25 μM of CX014442, and in untreated SupT1 cells. These ChIP-seq results were used to calculate gene category plots and distances. A) The relative proportion of integration sites categorized in four different genomic regions: silent genes (SG), intergenic regions (IR), regulatory elements (RE) and active genes (AG) in Jurkat cells from experiment A. B) The relative proportion of integration sites categorized in four different genomic regions in SupT1 cells from experiment A. C,D) Median distance in bp between integration sites and H3K36me3 (C) or H3K27ac (D) is shown for all experiments in Jurkat and SupT1 cells. All differences were statistically significant, ****p<0.0001.

Figure S6. Knockdown of LEDGF/p75 in SupT1 cells retargets integration out of active genes towards intergenic regions and silent genes.

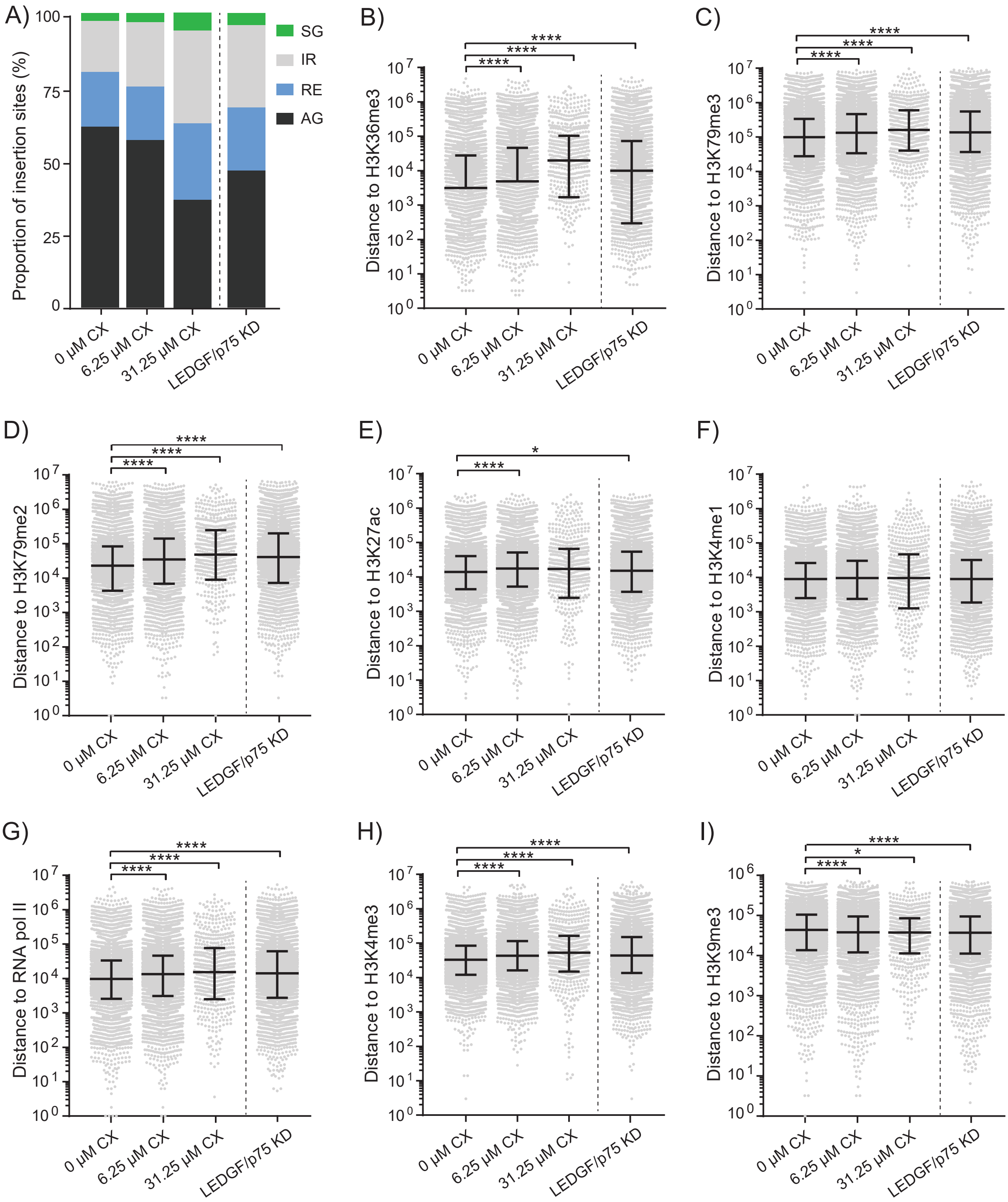

Figure S6. Knockdown of LEDGF/p75 in SupT1 cells retargets integration out of active genes towards intergenic regions and silent genes. Integration sites obtained in LEDGF/p75 knockdown SupT1 cells were compared with wild type SupT1 cells. Results are shown for one experiment in wild type SupT1 cells (experiment B) treated with 0, 6.25 or 31.25 µM of LEDGIN CX014442, and for LEDGF/p75 KD SupT1 cells (not treated with LEDGIN). A) Relative proportion of insertion sites found in silent genes (SG), intergenic regions (IR), regulatory elements (RE) and active genes (AG). B-I) The distance in base pairs (bp) between the integration site and certain features is plotted for each barcode. Each dot represents a unique integration site. Error bars represent median and interquartile range. The lower quartile is not plotted in case it equals '0'. Panels B-I plot the distance to: B) H3K36me3, C) H3K79me3, D) H3K79me2, E) H3K27ac, F) H3K4me1, G) RNAPII, H) H3K4me3 and I) H3K9me3. Statistical significance was calculated by the Kruskal-Wallis test, * $p<0.05$ and **** $p<0.0001$. KD; knockdown, bp; base pairs, RNAPII; RNA polymerase II.

Figure S7. Knockdown of LEDGF/p75 in Jurkat cells retargets integration out of active genes towards intergenic regions and silent genes.

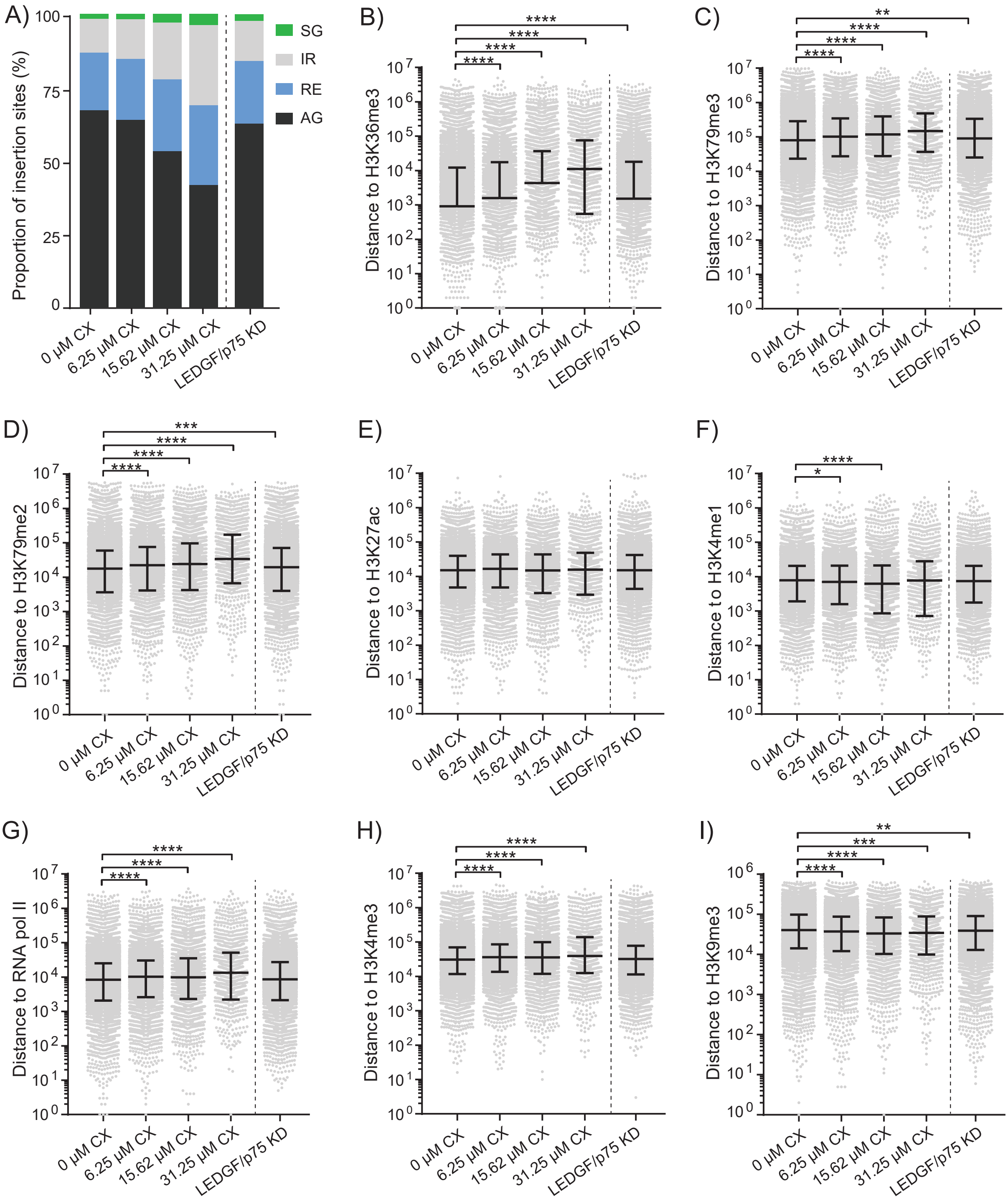

Figure S7. Knockdown of LEDGF/p75 in Jurkat cells retargets integration out of active genes towards intergenic regions and silent genes. Integration sites obtained in LEDGF/p75 knockdown Jurkat cells were compared with wild type Jurkat cells. Results are shown for one experiment in wild type Jurkat cells (experiment C) treated with 0, 6.25, 15.62 or 31.25 μM of LEDGIN CX014442, and for LEDGF/p75 KD Jurkat cells (not treated with LEDGIN). A) Relative proportion of insertion sites found in silent genes (SG), intergenic regions (IR), regulatory elements (RE) and active genes (AG). B-I) The distance in base pairs (bp) between the integration site and certain features is plotted for each barcode. Each dot represents a unique integration site. Error bars represent median and interquartile range. The lower quartile is not plotted in case it equals '0'. Panels B-I plot the distance to: B) H3K36me3, C) H3K79me3, D) H3K79me2, E) H3K27ac, F) H3K4me1, G) RNAPII, H) H3K4me3 and I) H3K9me3. Statistical significance was calculated by the Kruskal-Wallis test, * $p<0.05$ and **** $p<0.0001$. KD; knockdown, bp; base pairs, RNAPII; RNA polymerase II.

Figure S8. LEDGINs have no major effect on DNA methylation.

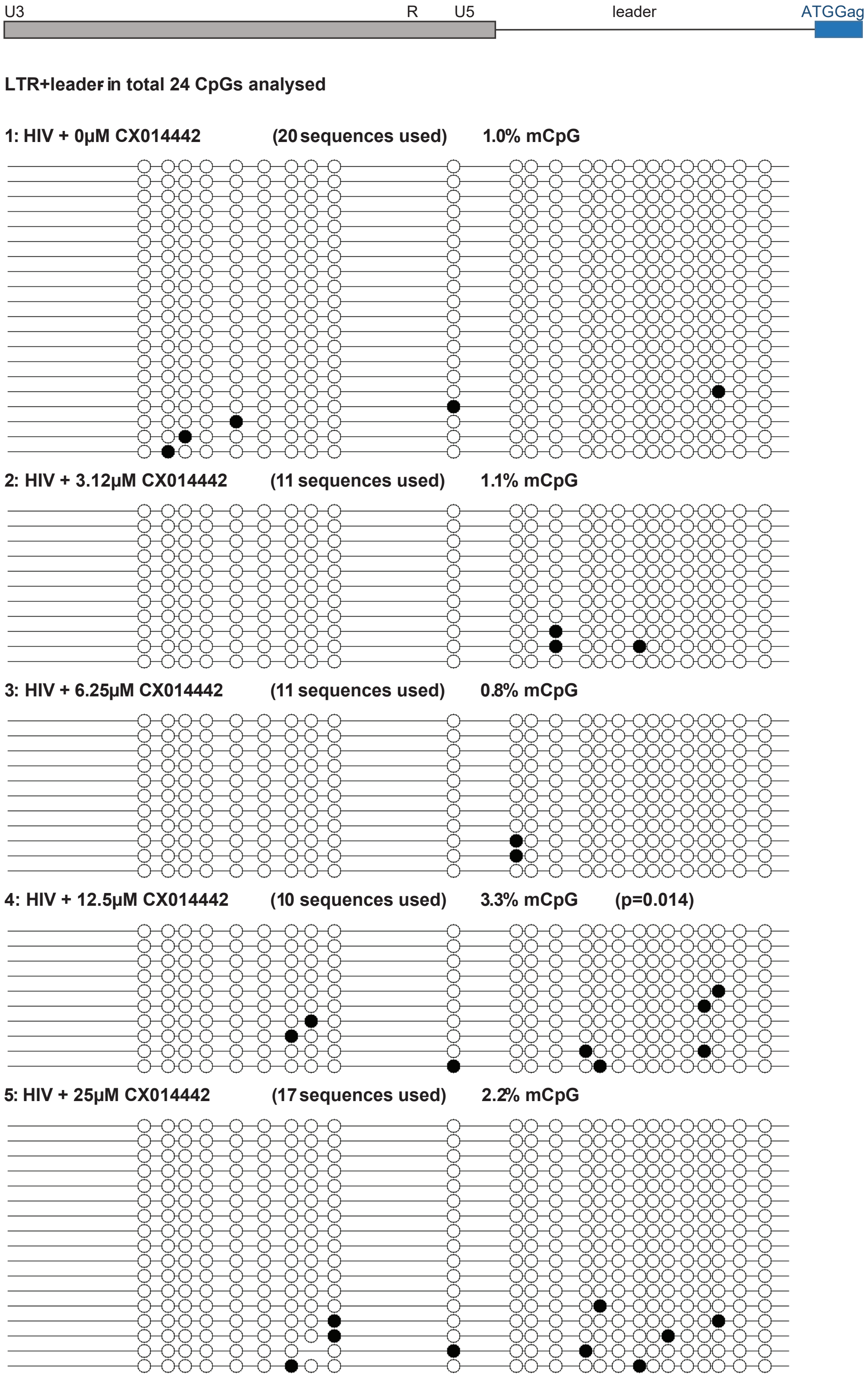

Figure S8. LEDGINs have no major effect on DNA methylation. The CpG methylation profiles of the HIV 5' LTR in non-treated (0 µM) and LEDGIN-treated (from 3.12 µM till 25 µM of CX014442) SupT1 cells. A schematic representation of the 5' LTR of the pOGH vector is shown. An analysis of the promoter molecules is shown as a linear array of open circles representing non-methylated CpG residues and closed circles representing methylated CpG residues. Each line represents one sequenced molecule of the 5' LTR. The methylation levels are presented as a mean percentage of methylated CpGs (mCpGs) in HIV-1 promoters. The p-values were calculated by the Chi-square test.

Figure S9. B-HIVE integration site analysis in GFP-sorted SupT1 cells.

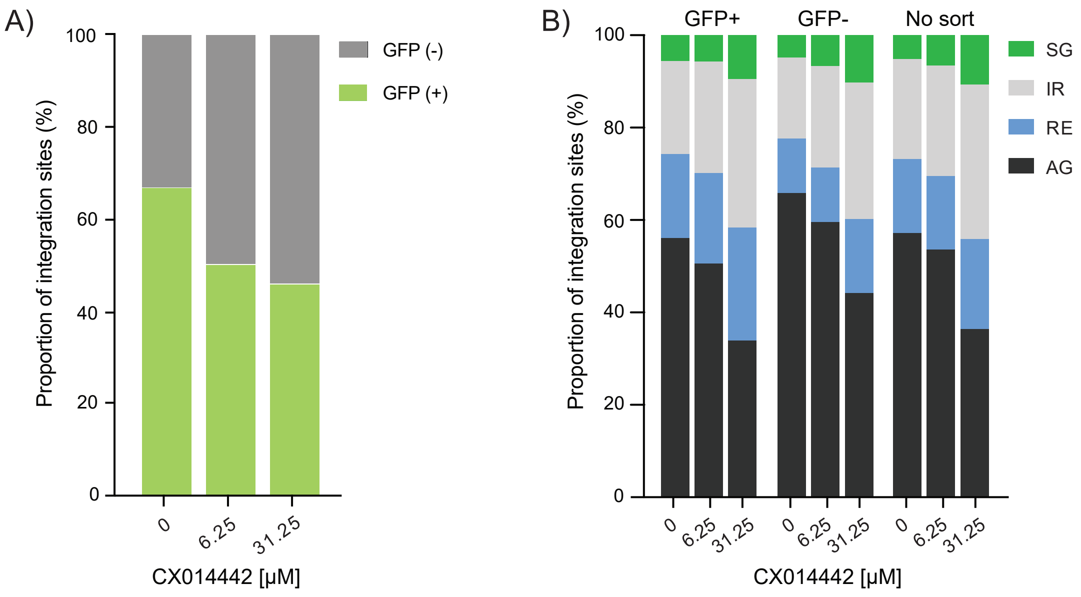

Figure S9. B-HIVE integration site analysis in GFP-sorted SupT1 cells. SupT1 cells were transduced with barcoded vector in the presence of various concentrations of LEDGIN CX014442 (0, 6.25 or 31.25 μM) and sorted based on GFP fluorescence four days post transduction. Sorted cells were cultured for two more weeks to determine DNA integration sites. A) The relative proportion of integration sites obtained in the different conditions in GFP positive (+, gray) and GFP negative (-, green) SupT1 cells. B) The relative proportion of integration sites categorized in four different genomic regions: silent genes (SG), intergenic regions (IR), regulatory elements (RE) and active genes (AG) for GFP(+), GFP(-) and unsorted SupT1 cells. GFP; Green Fluorescent Protein.

Figure S10. Comparison of ‘all’ integration sites versus ‘no RNA’ sites in Jurkat cells.

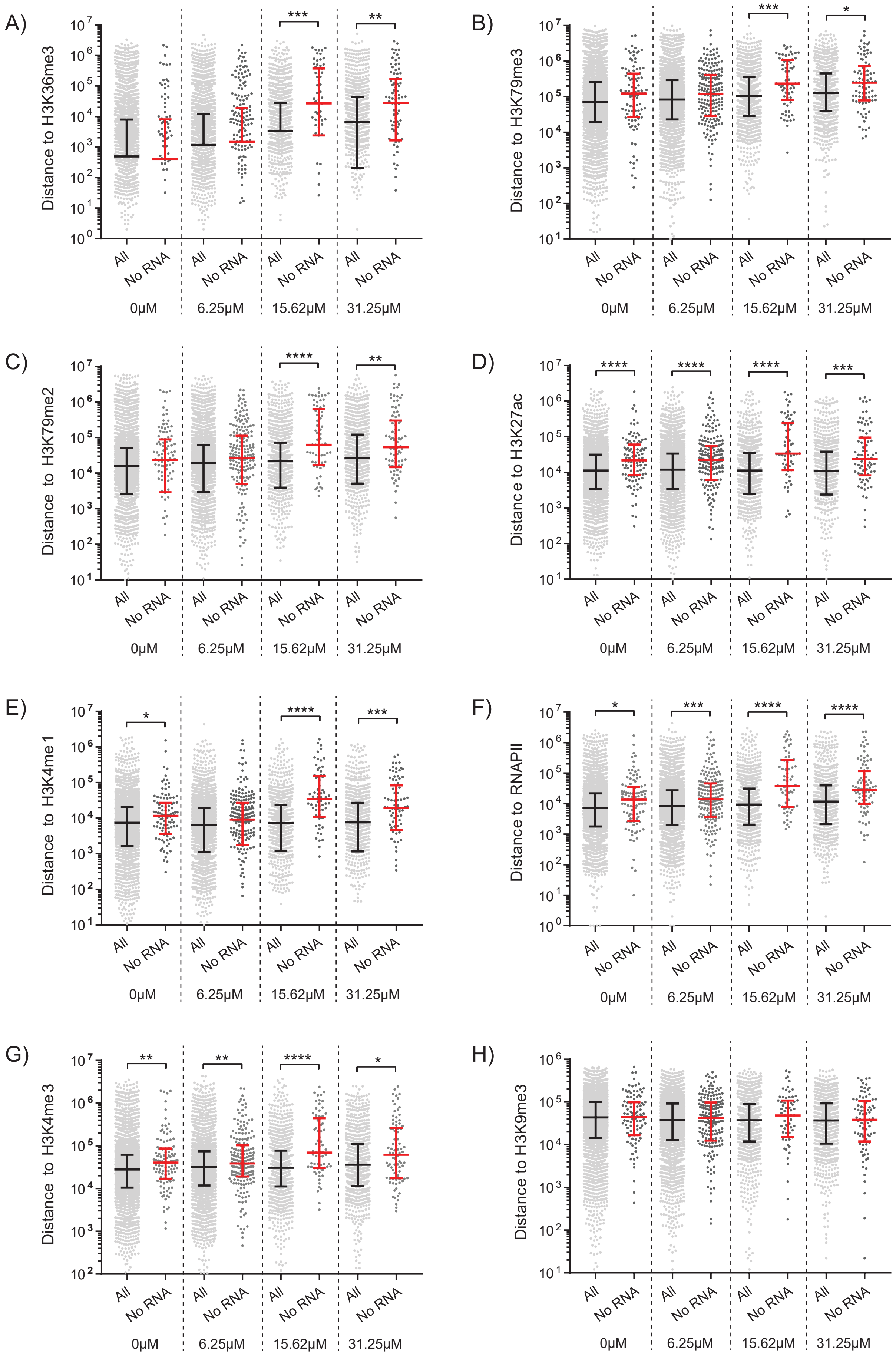

Figure S10. Comparison of 'all' integration sites versus 'no RNA' sites in Jurkat cells. The distance in base pairs (bp) of integration sites to certain features was determined for either 'all' retrieved insertion sites or the 'no RNA' sites in each condition (0, 6.25, 15.62 and 31.25 µM of CX014442). The distance (bp) is plotted on the y-axis for each barcode (dot) retrieved in one experiment in Jurkat cells. Error bars represent the median and interquartile ranges. Panels A-H plot the distance to: A) H3K36me3, B) H3K79me3, C) H3K79me2, D) H3K27ac, E) H3K4me1, F) RNAPII, G) H3K4me3 and H) H3K9me3. Statistical significance was calculated by the Kruskal-Wallis test, * $p<0.05$, ** $p<0.01$, *** $p<0.001$, **** $p<0.0001$. bp; base pairs, RNAPII; RNA polymerase II.

Figure S11. Gene categories for non-expressing integration sites.

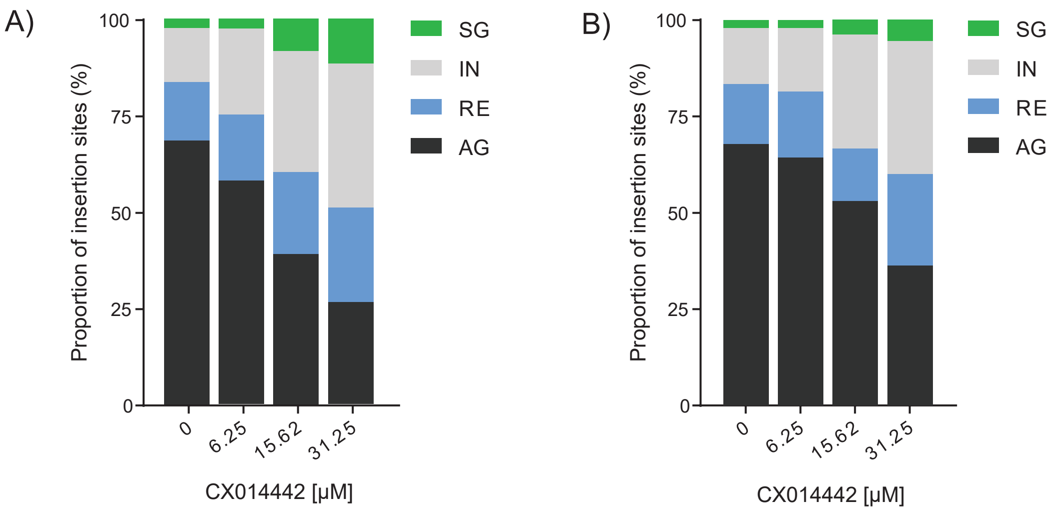

Figure S11. Gene categories for non-expressing integration sites. The relative proportion of non-expressing integration sites categorized in four different genomic regions: silent genes (SG), intergenic regions (IR), regulatory elements (RE) and active genes (AG) for SupT1 (A) and Jurkat (B) cells from experiment A.

Figure S12. Genes targeted by non-expressing proviruses in SupT1 cells.

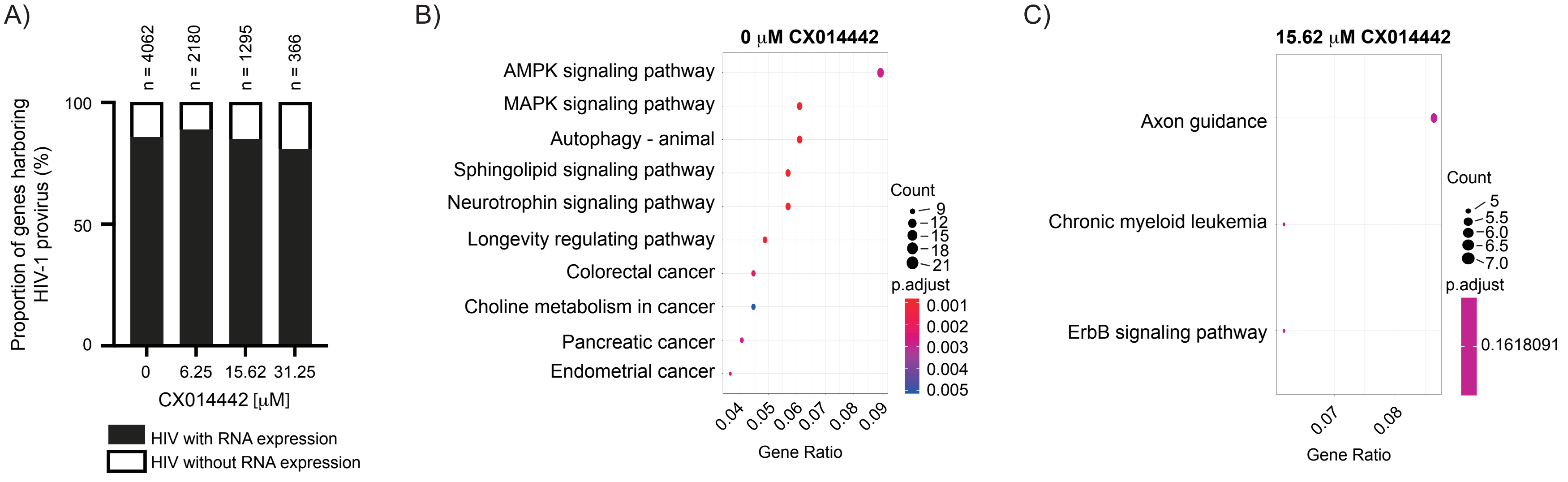

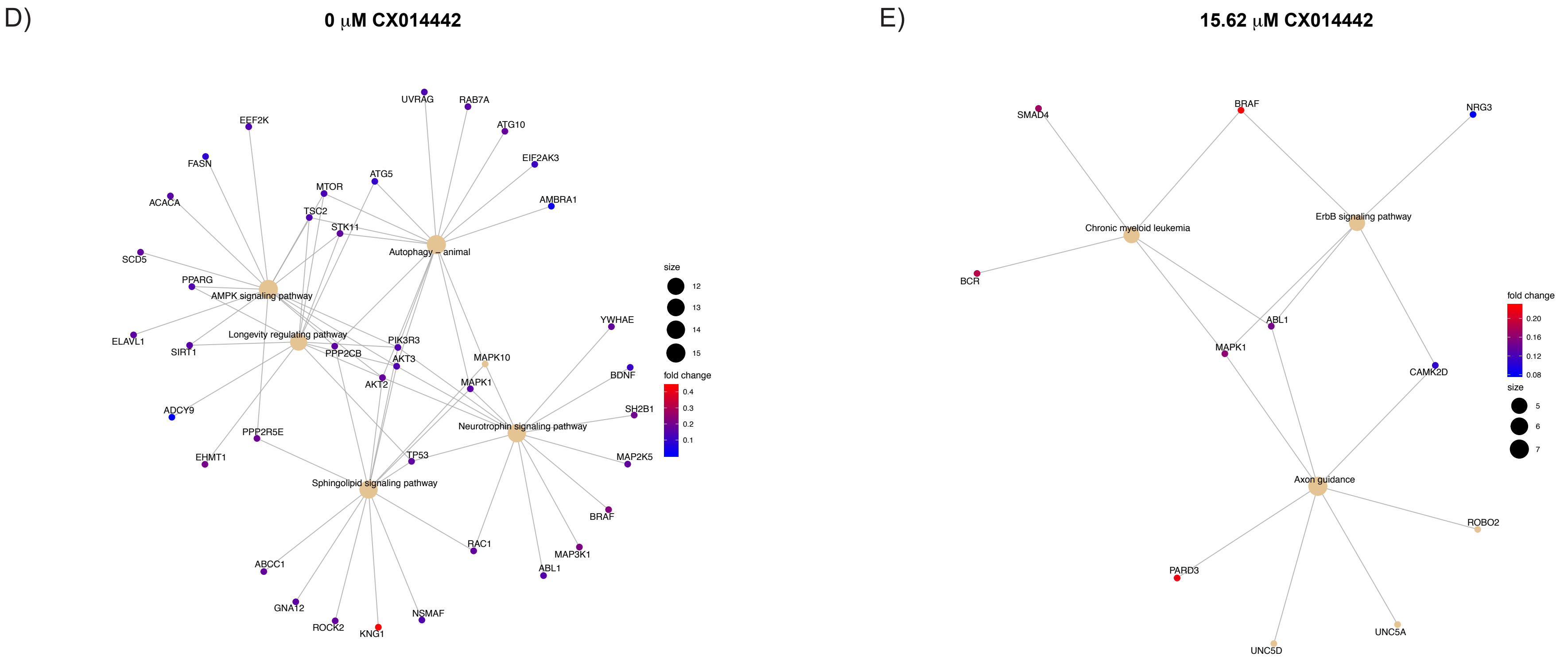

Figure S12. Genes targeted by non-expressing proviruses in SupT1 cells. A) Proportion of genes harboring transcriptionally active and non-active provirus in different genome categories after treatment with different concentrations of LEDGIN CX014442 (0, 6.25, 15.62 or 31.25 μM) in SupT1 cells. Black box: HIV with RNA expression; white box: HIV without RNA expression. B,C) Enriched KEGG-defined pathways from the genes harboring non-expressing HIV after treatment with 0 μM (B) and 15.62 μM (C) of LEDGIN CX014442. The size of the circle indicates the number of genes involved in each enriched pathway. The color of the circle indicates the adjusted P-value in each enriched pathway. D,E) The cnetplot illustrates the connection between the enriched pathways with significant adjusted P-values and the genes harboring non-expressing provirus after treatment with 0 μM (D) and 15.62 μM (E) of LEDGIN CX014442. The yellow node represents the selected enriched KEGG pathway. The size of the yellow node indicates the number of the genes involved in each selected enriched pathway. The color code in each node aside the annotated gene corresponds to the fold change in logarithm of endogenous gene expression after treatment with LEDGIN CX014442.

Figure S13. Genes targeted by non-expressing proviruses in Jurkat cells.

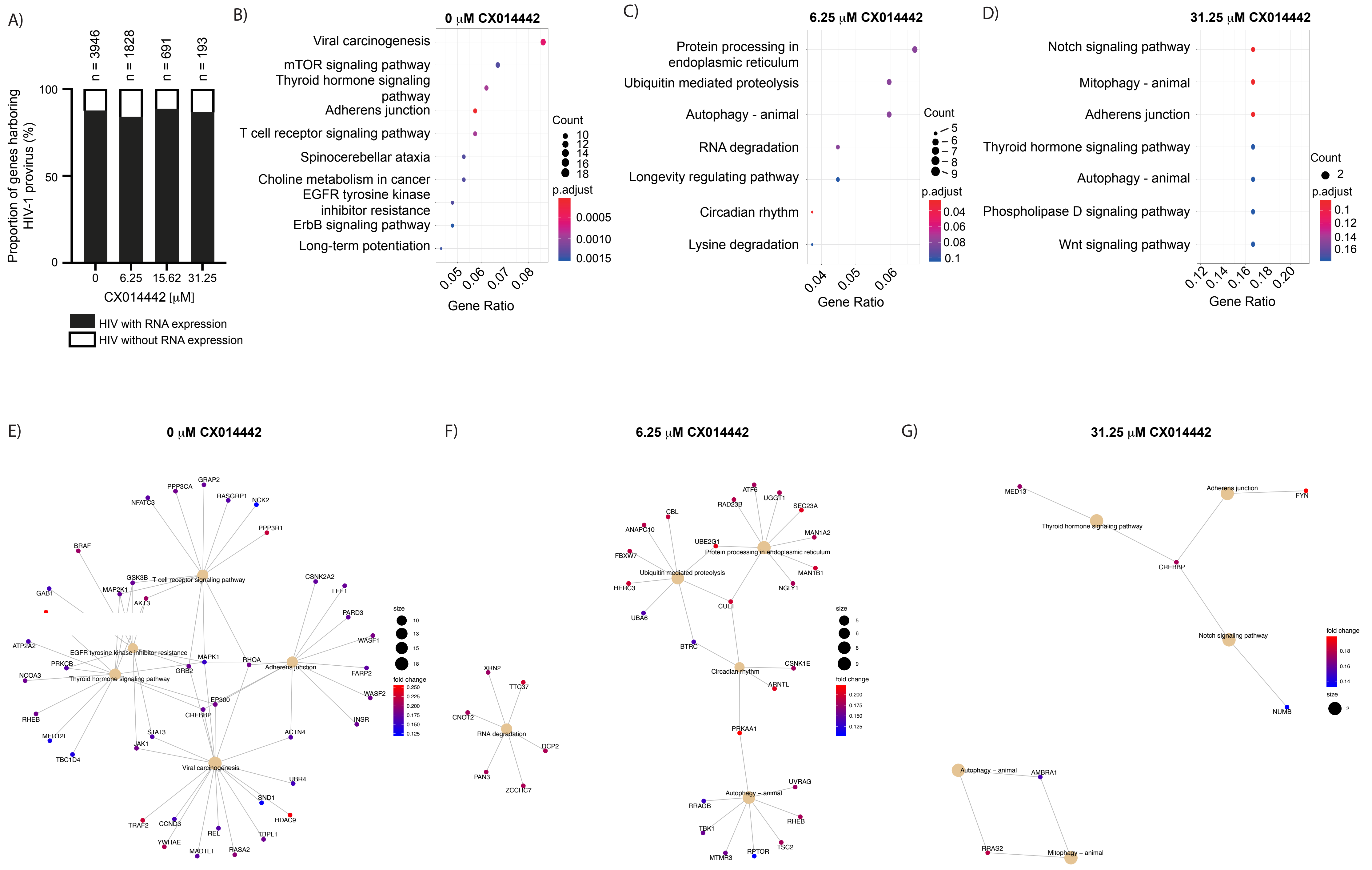

Figure S13. Genes targeted by non-expressing proviruses in Jurkat cells. A) Proportion of genes harboring transcriptionally active versus non-active provirus in different genome categories after treatment with different concentrations of LEDGIN CX014442 (0, 6.25, 15.62 or 31.25 µM) in Jurkat cells. Black box: HIV with RNA expression; white box: HIV without RNA expression. B-D) Enriched KEGG-defined pathways from the genes harboring non-expressing HIV after treatment with 0 µM (B), 6.25 µM (C) or 31.25 µM (D) of LEDGIN CX014442. The size of the circle indicates the number of genes involved in each enriched pathway. The color of the circle indicates the adjusted P-value in each enriched pathway. E-G) The cnetplot illustrates the connection between the enriched pathways with significant adjusted P-values and the genes harboring non-expressing provirus after treatment with 0 µM (E), 6.25 µM (F) or 31.25 µM (G) of LEDGIN CX014442. The yellow node represents the selected enriched KEGG pathway. The size of the yellow node indicates the number of the genes involved in each selected enriched pathway. The color code in each node aside the annotated gene corresponds to the fold change in logarithm of endogenous gene expression after treatment with LEDGIN CX014442.

Figure S14. Comparison between ‘all sites’ and ‘high expressors’ in three experiments.

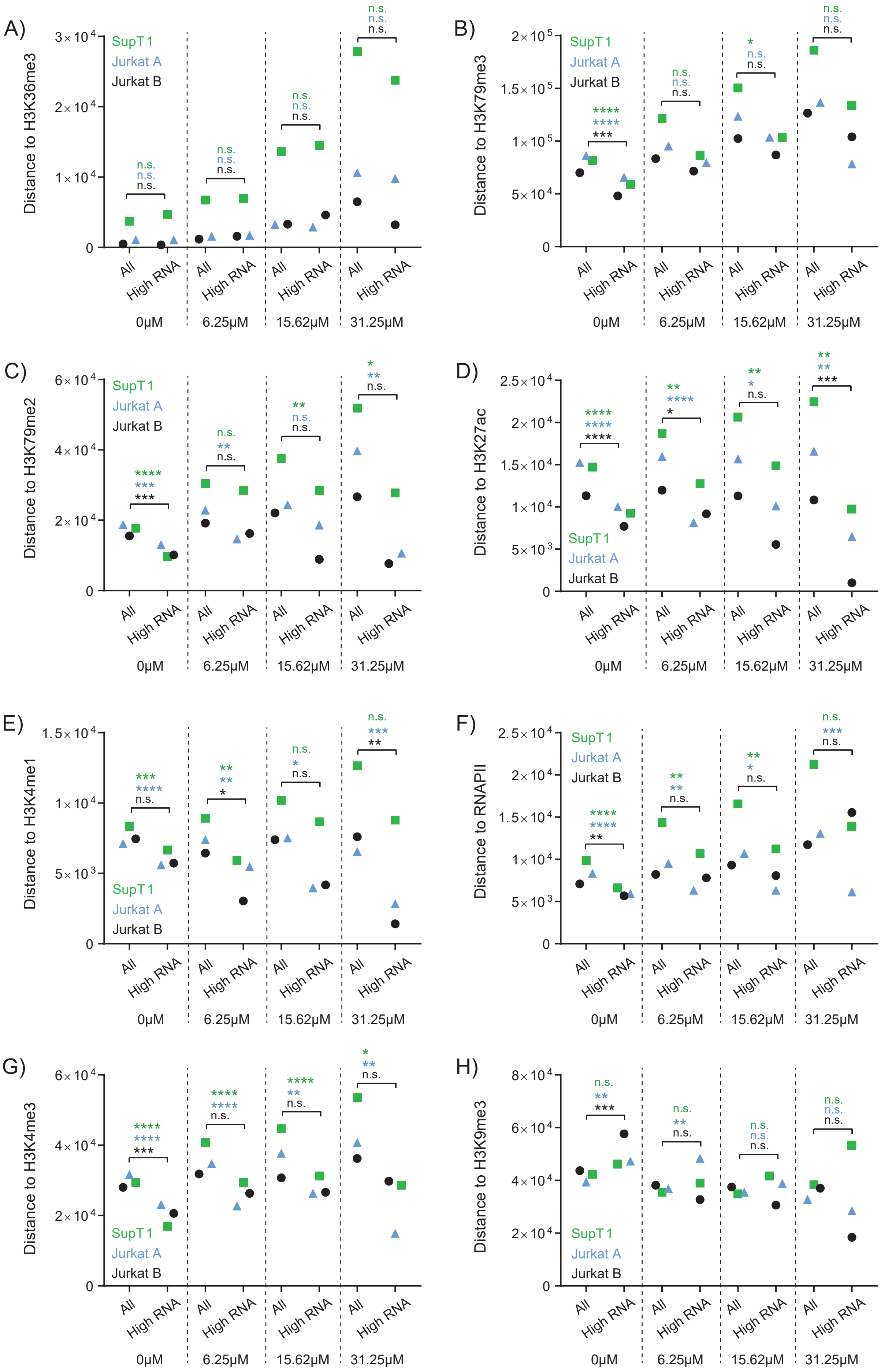

Figure S14. Comparison between 'all' sites and 'high expressors' in three experiments. The distance in base pairs (bp) of integration sites to certain features was determined for all retrieved insertion sites or the 10 % highest RNA expressing sites in each condition (0, 6.25, 15.62 or 31.25 µM of CX014442). The median distance (bp) is plotted on the y-axis for two independent experiments in Jurkat cells (experiment A blue, experiment B black) and one experiment in SupT1 cells (green). Panels A-H plot the distance to: A) H3K36me3, B) H3K79me3, C) H3K79me2, D) H3K27ac, E) H3K4me1, F) RNAPII, G) H3K4me3 and H) H3K9me3. Statistical significance was calculated by the Kruskal-Wallis test, * $p<0.05$, ** $p<0.01$, *** $p<0.001$, **** $p<0.0001$. bp; base pairs, RNAPII; RNA polymerase II.
